# Supplementary material for: Ultraviolet A light effectively reduces bacteria and viruses including coronavirus
Source: PLoS One. 2020 Jul 16;15(7):e0236199. doi: 10.1371/journal.pone.0236199 (PMC7365468; doi:10.1371/journal.pone.0236199)

**S1 Figure.** A) Customized side-emitting borosilicate light rod used for broad-band UVA experiments. B) Narrow-band UVA LED light configuration. C) UVA light application in mice for *in vivo* safety determination. Right side: mouse undergoing colonic UVA therapy up to splenic flexure under anesthesia. Left side: control mouse exposed to unlit rod.


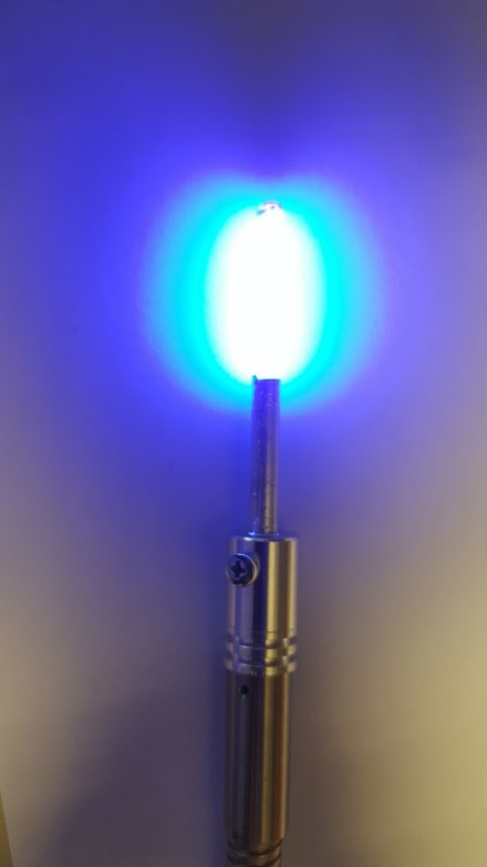


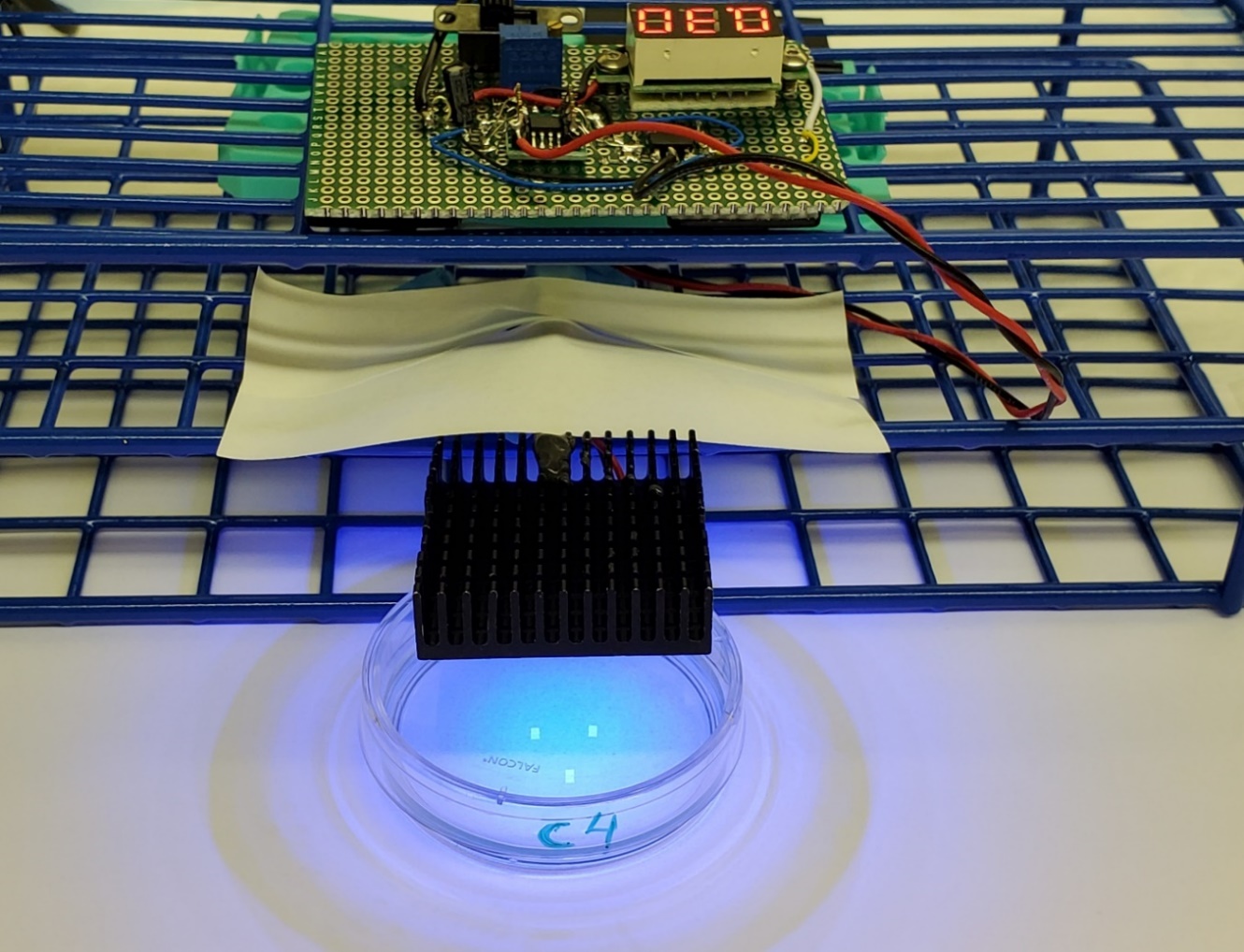


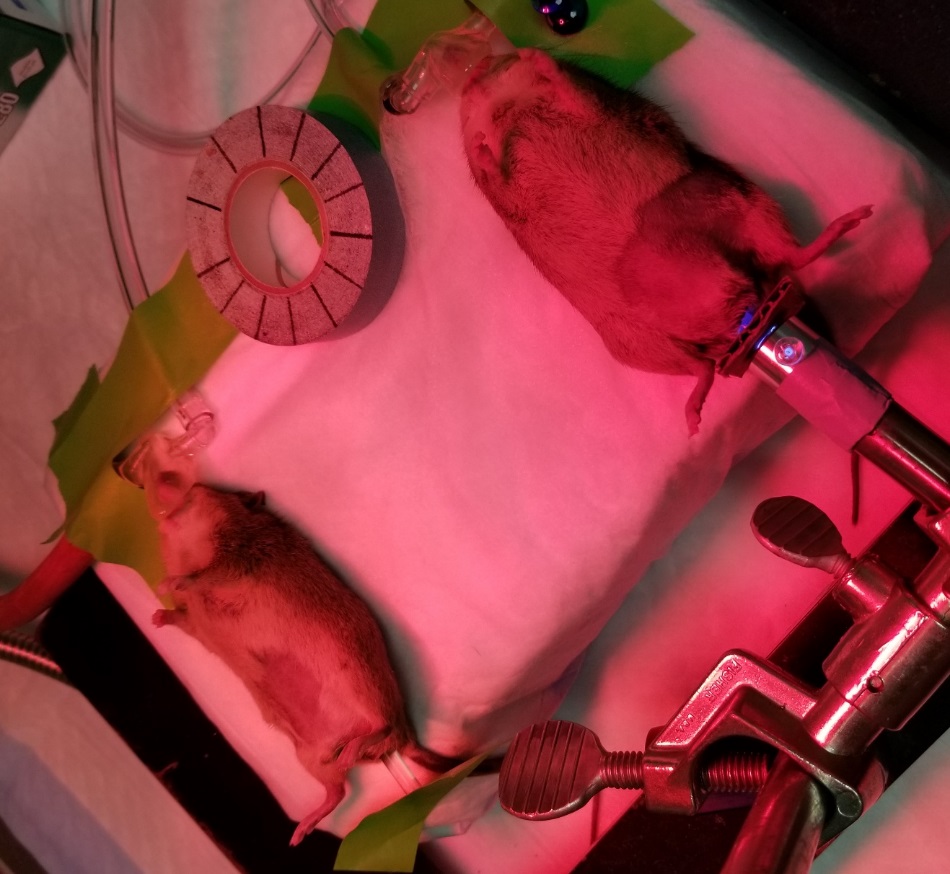

Supplement: S1 Fig — A) Customized side-emitting borosilicate light rod used for broad-band UVA experiments. B) Narrow-band UVA LED light configuration. C) UVA light application in mice for in vivo safety determination. Right side: mouse undergoing colonic UVA therapy up to splenic flexure under anesthesia. Left side: control mouse exposed to unlit rod. (DOCX) [file pone.0236199.s001.docx]
