# Supplementary material for: Ultraviolet A light effectively reduces bacteria and viruses including coronavirus
Source: PLoS One. 2020 Jul 16;15(7):e0236199. doi: 10.1371/journal.pone.0236199 (PMC7365468; doi:10.1371/journal.pone.0236199)

**S2 Fig.** Effects of NB-UVA exposure on HeLa cells transfected with group B coxsackievirus. A) 24 hours after transfection: Reduced number of adherent cells in UVA-unexposed plates (left panel, percent dead cells in supernatant=67.5±11.0%) compared with UVA-exposed plates (right panel, percent dead cells in supernatant=16.1±5.8%) (P=0.002). (Magnification=4x, overlay of green light and bright field). B) 48 hours after transfection: Left panel shows no remaining live cells (unexposed to UVA). Right panel shows survival of UVA-exposed cells (Magnification=4x, overlay of green light and bright field).


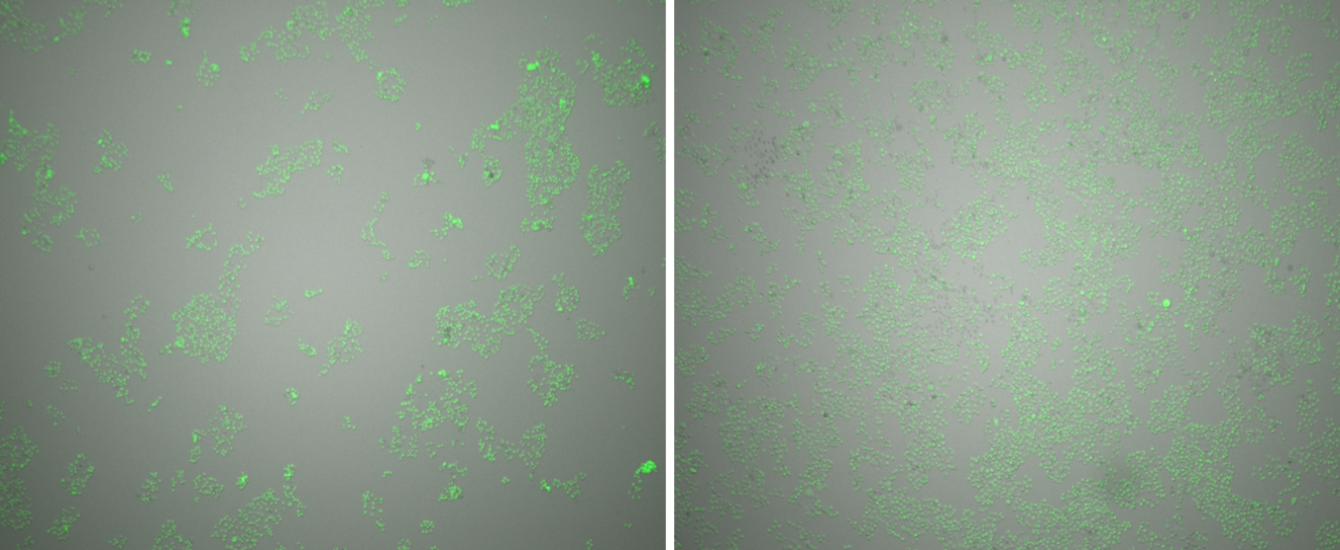


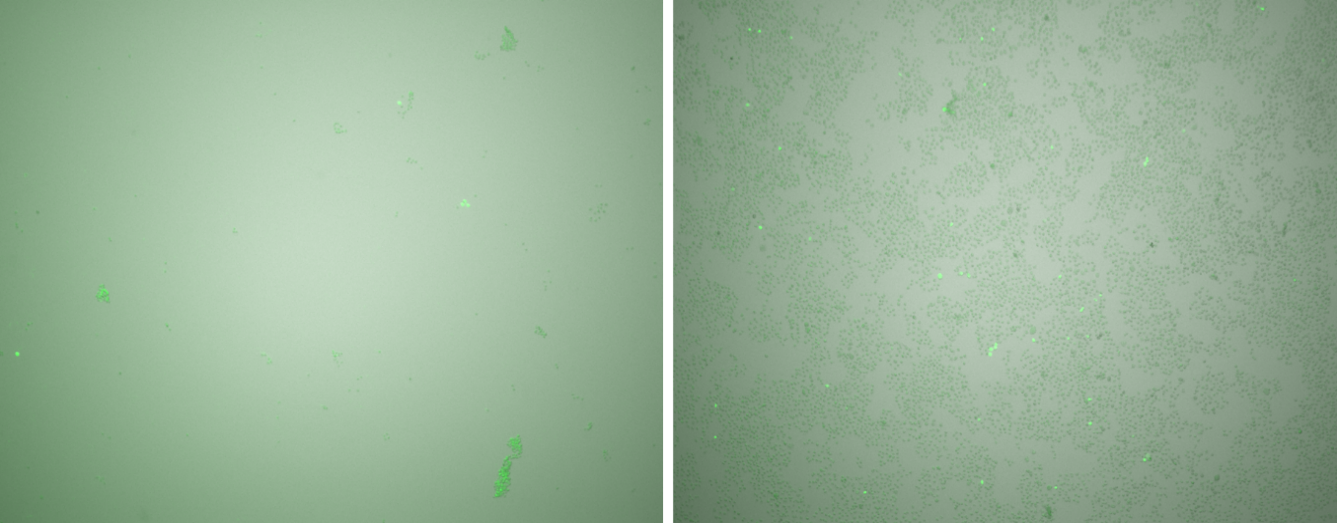

Supplement: S2 Fig — A) 24 hours after transfection: Reduced number of adherent cells in UVA-unexposed plates (left panel, percent dead cells in supernatant = 67.5±11.0%) compared with UVA-exposed plates (right panel, percent dead cells in supernatant = 16.1±5.8%) (P = 0.002). (Magnification = 4x, overlay of green light and bright field). (DOCX) [file pone.0236199.s002.docx]
