# Supplementary material for: Ultraviolet A light effectively reduces bacteria and viruses including coronavirus
Source: PLoS One. 2020 Jul 16;15(7):e0236199. doi: 10.1371/journal.pone.0236199 (PMC7365468; doi:10.1371/journal.pone.0236199)

**S3 Fig.** Effects of NB-UVA exposure on alveolar (A549) cells transfected with EGFP-CVB Transfected alveolar cells treated with NB UVA exhibit less viral EGFP signals (left panel) when compared to transfected alveolar cells not treated with UVA (right panel) (Magnification=4x, overlay of green light and bright field).


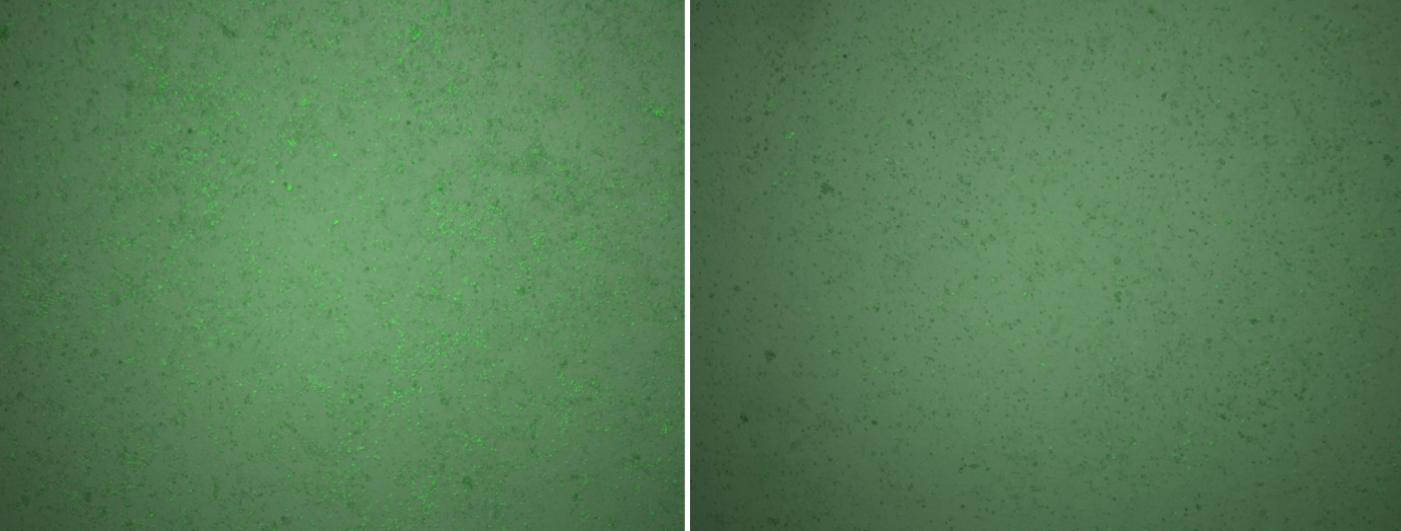

Supplement: S3 Fig — Effects of NB-UVA exposure on alveolar (A549) cells transfected with EGFP-CVB Transfected alveolar cells treated with NB UVA exhibit less viral EGFP signals (left panel) when compared to transfected alveolar cells not treated with UVA (right panel) (Magnification = 4x, overlay of green light and bright field). (DOCX) [file pone.0236199.s003.docx]
