# Supplementary material for: Ultraviolet A light effectively reduces bacteria and viruses including coronavirus
Source: PLoS One. 2020 Jul 16;15(7):e0236199. doi: 10.1371/journal.pone.0236199 (PMC7365468; doi:10.1371/journal.pone.0236199)
Supplement: S1 Table — (DOCX) [file pone.0236199.s004.docx]

**S1 Table.** *In vitro* exposure of pathogens to UVA, including growth conditions, intensity, and duration of UVA exposures

| **Microbial strains** | **Liquid broth*** | **Solid medium (agar-based plates)** | **Incubation Temperature (°C)** | **Atmosphere** | **Duration of initial incubation (hours)** | **Duration of incubation prior to UVA exposure (hours)** | **Intensity of UVA light (µW/cm^2^)** | **Duration of UVA exposure (minutes)** |
| --- | --- | --- | --- | --- | --- | --- | --- | --- |
| *Candida albicans* (Robin) Berkhout ATCC® 10231™ | Sabouraud Dextrose | Sabouraud Dextrose | 24 to 26 | Aerobic | 16 to 24 | 4 to 6 | 1700 | 20, 40 and 60 |
| *Clostridioides difficile* (Prevot) Lawson et al. ATCC® 700057™ | Brain Heart Infusion | Reinforced Clostridial | 36 to 37 | Anaerobic | 24 to 48 | 6 to 8 | 2000 | 20, 40 and 60 |
| *Enterococcus faecalis* ATCC® 29212™ | Brain Heart Infusion | Trypticase Soy Agar with 5% Sheep Blood | 36 to 37 | Aerobic | 18 to 24 | 4 to 6 | 2400 | 20, 40 and 60 |
| *Escherichia coli* GFP ATCC® 25922 GFP™ | Luria Bertani | Luria Bertani | 36 to 37 | Aerobic | 16 to 24 | 2 to 3 | 1300 | 20, 40 and 60 |
| *Escherichia coli* - clinical isolate | Luria Bertani | Luria Bertani | 36 to 37 | Aerobic | 16 to 24 | 2 to 3 | 1100 to 1300 | 20, 40, 60 and 80 |
| *Klebsiella pneumoniae* ATCC® BAA-1705™ | Luria Bertani | Luria Bertani | 36 to 37 | Aerobic | 16 to 24 | 2 to 3 | 1300 | 20, 40 and 60 |
| *Proteus mirabilis* ATCC® 29906™ | Luria Bertani | Hectoen Enteric | 36 to 37 | Aerobic | 16 to 24 | 2 to 3 | 2400 | 20, 40 and 60 |
| *Pseudomonas aeruginosa* ATCC® 15442™ | Luria Bertani | Luria Bertani | 36 to 37 | Aerobic | 16 to 24 | 2 to 3 | 3500 | 20, 40 and 60 |
| *Staphylococcus epidermidis* (Winslow and Winslow) Evans ATCC® 14990™ | Tryptic Soy Broth | Trypticase Soy Agar with 5% Sheep Blood | 36 to 37 | Aerobic | 24 to 48 | 3 to 5 | 2150 | 20, 40 and 60 |
| *Streptococcus pyogenes* Rosenbach ATCC® 19615™ | Tryptic Soy Broth | Trypticase Soy Agar with 5% Sheep Blood | 36 to 37 | Anaerobic | 24 to 48 | 3 to 5 | 1800 | 20,40 and 60 |

* A single colony was cultured in 5 ml broth
