# Supplementary material for: Ultraviolet A light effectively reduces bacteria and viruses including coronavirus
Source: PLoS One. 2020 Jul 16;15(7):e0236199. doi: 10.1371/journal.pone.0236199 (PMC7365468; doi:10.1371/journal.pone.0236199)
Supplement: S2 Table — (DOCX) [file pone.0236199.s005.docx]

**S2_Table.** Effect of NB-UVA light on bacterial colony diameter based on time exposure across varying intensities

| **Microorganism** | **UVA Intensity (µW/cm^2^)** | **Group** | **Baseline** | **20 min** | **P value** | **40 min** | **P value** |
| --- | --- | --- | --- | --- | --- | --- | --- |
|  |  |  | **Mean CFU Ø (mm)** | **Mean CFU Ø (mm)** |  | **Mean CFU Ø (mm)** |  |
| *Escherichia coli* GFP | 500 | Exposed | 1.59 | 1.57 | 0.81 | 1.59 | 0.89 |
|  |  | Control | 1.59 | 1.59 |  | 1.57 |  |
| *Escherichia coli* GFP | 1000 | Exposed | 1.76 | 1.67 | 0.08 | 1.38 | 0.01 |
|  |  | Control | 1.76 | 1.73 |  | 1.73 |  |
| *Escherichia coli* GFP | 2000 | Exposed | 1.71 | 1.38 | <0.001 | 0.93 | <0.001 |
|  |  | Control | 1.71 | 1.70 |  | 1.72 |  |
| *Escherichia coli* GFP | 3000 | Exposed | 1.83 | 0.25 | <0.01 | 0.00 | <0.01 |
|  |  | Control | 1.83 | 1.80 |  | 1.69 |  |
| *Pseudomonas aeruginosa* | 500 | Exposed | 0.90 | 0.78 | 0.16 | 0.77 | 0.35 |
|  |  | Control | 0.90 | 0.92 |  | 0.84 |  |
| *Pseudomonas aeruginosa* | 1000 | Exposed | 1.08 | 0.79 | <0.01 | 0.65 | <0.001 |
|  |  | Control | 1.08 | 1.06 |  | 1.03 |  |
| *Pseudomonas aeruginosa* | 2000 | Exposed | 1.05 | 0.69 | <0.001 | 0.63 | <0.001 |
|  |  | Control | 1.05 | 1.04 |  | 1.00 |  |
| *Pseudomonas aeruginosa* | 3000 | Exposed | 1.33 | 0.88 | 0.01 | 0.79 | <0.01 |
|  |  | Control | 1.33 | 1.29 |  | 1.30 |  |
