# Supplementary figures and images for: Ultraviolet A light effectively reduces bacteria and viruses including coronavirus
Source: PLoS One. 2020 Jul 16;15(7):e0236199. doi: 10.1371/journal.pone.0236199 (PMC7365468; doi:10.1371/journal.pone.0236199)

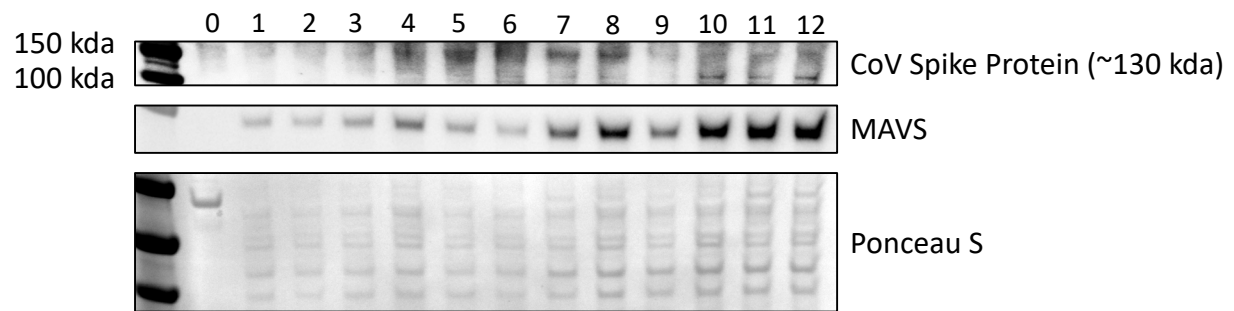

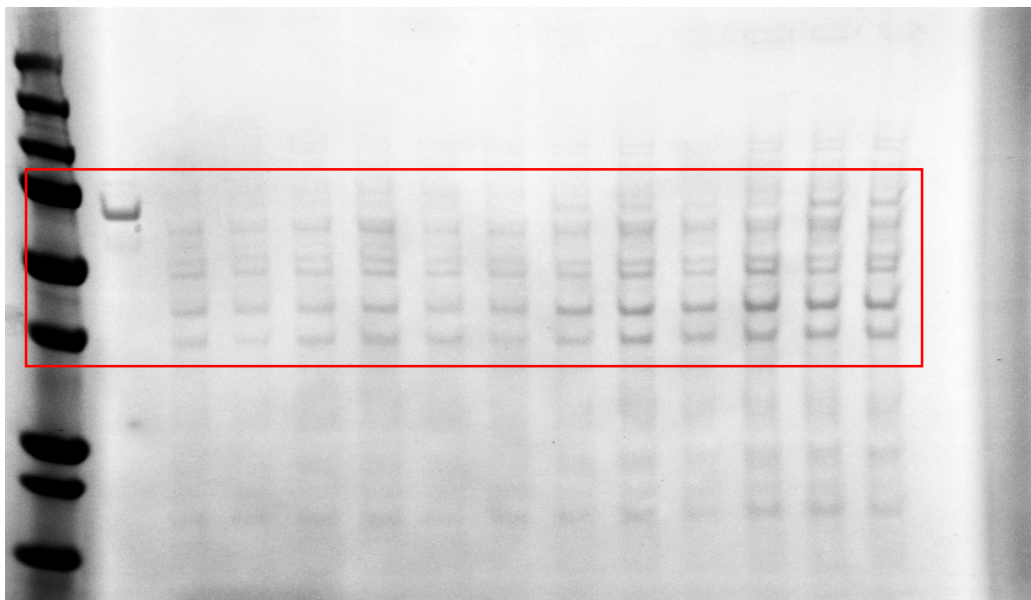

Ponceau S

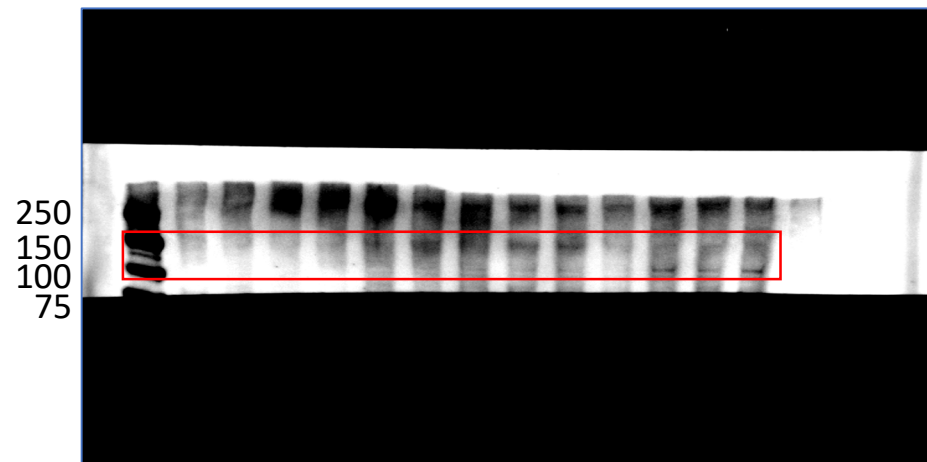

CoV Spike Protein (~130 kda)

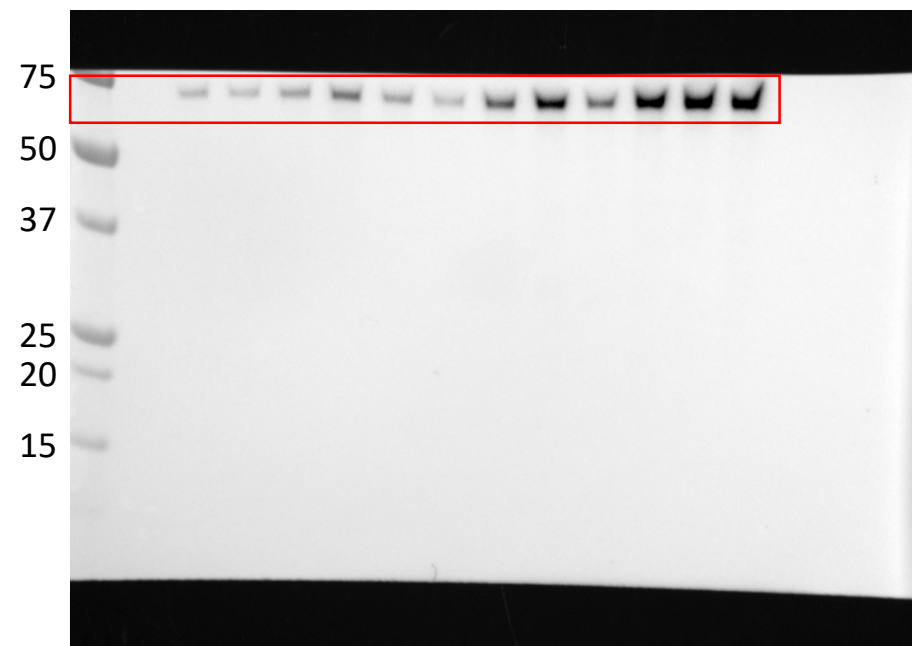

MAVS (~75 kda)

Supplement: S1 Raw Images — (PDF) [file pone.0236199.s006.pdf]
