# Supplementary material for: Correction: Ultraviolet A light effectively reduces bacteria and viruses including coronavirus
Source: PLoS One. 2020 Aug 11;15(8):e0237782. doi: 10.1371/journal.pone.0237782 (PMC7418994; doi:10.1371/journal.pone.0237782)
Supplement: S1 File — (PDF) [file pone.0237782.s001.pdf]

PLOS ONE

RESEARCH ARTICLE

Ultraviolet a light effectively reduces bacteria and viruses including coronavirus

Ali Rezaie[1]*, Gabriela G. S. Leite[1], Gil Y. Melmed[2], Ruchi Mathur[1], Maria Jesus Villanueva-Millan[1], Gonzalo Parodi[1], Jon Sin[3], Juliana F. Germano[3], Walter Morales[1], Stacy Weitsman[1], Seung Young Kim[4], Jae Ho Park[5], Siamak Sakhaie[6], Mark Pimentel[1]

1 Medically Associated Science and Technology (MAST) Program, Cedars-Sinai Medical Center, Los Angeles, California, United States of America, **2** Inflammatory Bowel and Immunobiology Research Institute, Cedars-Sinai, Los Angeles, California, United States of America, **3** Cedars-Sinai Heart Institute, Cedars-Sinai, Los Angeles, California, United States of America, **4** Division of Gastroenterology, Department of Internal Medicine, Korea University Ansan Hospital, Ansan, South Korea, **5** Division of Gastroenterology, Department of Internal Medicine, Ulsan University Hospital, Ulsan, South Korea, **6** Australian Clinical Labs, Melbourne, Australia

* ali.rezaie@cshs.org

Abstract

Antimicrobial-resistant and novel pathogens continue to emerge, outpacing efforts to contain and treat them. Therefore, there is a crucial need for safe and effective therapies. Ultraviolet-A (UVA) phototherapy is FDA-approved for several dermatological diseases but not for internal applications. We investigated UVA effects on human cells *in vitro*, mouse colonic tissue *in vivo*, and UVA efficacy against bacteria, yeast, coxsackievirus group B and coronavirus-229E. Several pathogens and virally transfected human cells were exposed to a series of specific UVA exposure regimens. HeLa, alveolar and primary human tracheal epithelial cell viability was assessed after UVA exposure, and 8-Oxo-2'-deoxyguanosine was measured as an oxidative DNA damage marker. Furthermore, wild-type mice were exposed to intracolonic UVA as an *in vivo* model to assess safety of internal UVA exposure. Controlled UVA exposure yielded significant reductions in *Pseudomonas aeruginosa*, *Klebsiella pneumoniae*, *Escherichia coli*, *Enterococcus faecalis*, *Clostridioides difficile*, *Streptococcus pyogenes*, *Staphylococcus epidermidis*, *Proteus mirabilis* and *Candida albicans*. UVA-treated coxsackievirus-transfected HeLa cells exhibited significantly increased cell survival compared to controls. UVA-treated coronavirus-229E-transfected tracheal cells exhibited significant coronavirus spike protein reduction, increased mitochondrial antiviral-signaling protein and decreased coronavirus-229E-induced cell death. Specific controlled UVA exposure had no significant effect on growth or 8-Oxo-2'-deoxyguanosine levels in three types of human cells. Single or repeated *in vivo* intraluminal UVA exposure produced no discernible endoscopic, histologic or dysplastic changes in mice. These findings suggest that, under specific conditions, UVA reduces various pathogens including coronavirus-229E, and may provide a safe and effective treatment for infectious diseases of internal viscera. Clinical studies are warranted to further elucidate the safety and efficacy of UVA in humans.

Check for updates

OPEN ACCESS

Citation: Rezaie A, Leite GGS, Melmed GY, Mathur R, Villanueva-Millan MJ, Parodi G, et al. (2020) Ultraviolet a light effectively reduces bacteria and viruses including coronavirus. PLoS ONE 15(7): e0236199. https://doi.org/10.1371/journal.pone.0236199

Editor: Michael R. Hamblin, Massachusetts General Hospital, UNITED STATES

Received: May 11, 2020

Accepted: June 30, 2020

Published: July 16, 2020

Copyright: © 2020 Rezaie et al. This is an open access article distributed under the terms of the Creative Commons Attribution License, which permits unrestricted use, distribution, and reproduction in any medium, provided the original author and source are credited.

Data Availability Statement: All relevant data are within the paper and its Supporting Information files.

Funding: This project was partly funded by a grant to AR (award number 2017-494) from the Kenneth Rainin Foundation. It provided salary support for AR and GL. https://krfoundation.org/. The funder had no role in study design, data collection and analysis, decision to publish, or preparation of the manuscript. SS is an employee of Australian Clinical Labs. Australian Clinical Labs had no role in

study design, data collection and analysis, decision to publish, or preparation of the manuscript.

Competing interests: Cedars-Sinai Medical Center has a licensing agreement with Aytu BioSciences. Cedars-Sinai has a patent on internal UV therapy, inventors: AR, MP, GM, RM and GL. SS is an employee of Australian Clinical Labs. The authors declare no other financial conflicts of interest. This does not alter our adherence to PLOS ONE policies on sharing data and materials.

Introduction

Infections have been the primary cause of human morbidity and mortality throughout recorded history. Antimicrobial-resistant and novel pathogens continue to emerge, outpacing efforts to contain and treat them. In December 2019, a novel severe acute respiratory syndrome coronavirus 2 (SARS-CoV-2) outbreak was reported [1] and has rapidly become a global pandemic. Safe and effective therapies for treatment-resistant and novel pathogens are urgently needed.

Ultraviolet (UV) light has long been known to exhibit antimicrobial effects. UVC (100–280 nm) [2, 3] is widely used to decontaminate environmental surfaces [4], but has harmful effects on human DNA [5]. External UVA (315-400nm) [2, 3] and UVB (280–315 nm) [2, 3] are FDA-approved for dermatologic indications including psoriasis, eczema and skin lymphoma [6–9]. Among these spectra, UVA, which composes 90–98% of the UV radiation in terrestrial sunlight, appears least damaging to mammalian cells [3, 10]. Recent advances in light emitting diodes (LEDs) make it feasible to apply light to internal organs [11].

Presently, there are no studies exploring the internal application of UVA light for bacterial or viral infections. Here, under specific conditions including distance, wavelength, intensity and time, we assess UVA efficacy against bacterial, fungal, and viral pathogens, including group B coxsackievirus and coronavirus-229E. We also evaluate the effects of therapeutic and supratherapeutic UVA exposure *in vitro* on three human cell types. Furthermore, we assess the effects of intraluminal UVA exposure *in vivo* in the first animal model of internal UVA therapy.

Materials and methods

Effects of UVA light on common opportunistic microbes in culture

Bacterial and yeast preparations. Bacteria and yeast were grown in appropriate liquid culture media and conditions (detailed in S1 Table). Primary cultures were used to inoculate solid microbial agar and isolate single colony forming units (CFU). Liquid cultures were prepared from a single CFU of each microbe to guarantee purity. Cultures were incubated (S1 Table) until they reached the McFarland standard of 0.5 [12] and 1000 μL of the liquid culture was transferred into each of two 1.7 mL micro-centrifuge sterile tubes. A 100 μL aliquot from each tube was serially diluted and plated on solid microbial medium to determine baseline CFU/mL (S1 Table), and UVA light was applied to the remainder.

UVA light against bacteria and yeast. UVA effects were assessed using both broad band (BB) and narrow band (NB) wavelength spectra. For BB assessments (peak wavelength ~345nm), a mercury vapor lamp (Asahi Max 303, Asahi Spectra Co., Tokyo, Japan) was used to transmit light *via* a borosilicate rod etched with diluted sulfuric acid, sodium bifluoride, barium sulfate and ammonium bifluoride (Armour, NJ). For NB experiments, an array of LEDs (peak wavelength 343±3nm, with full width at half maximum of 5nm) mounted on an aluminum heatsink (Seoul Viosys, Gyeonggi-Do, South Korea) (S1 Fig) was used. Wavelengths were confirmed by spectrometry (Flame UV-VIS, Ocean Optics, FL) and UV meters (SDL470 and UV510 UV, Extech, NH) (S1 Fig).

For the BB-UVA experiments, the sterilized rod was placed through the caps of 1.7mL tubes. An identical unlit rod was placed into control tubes. After incubation, CFU/mL were determined by serial dilutions of aliquots and measured using a Scan 300 Automatic Colony Counter (Interscience, Woburn, MA). This process was repeated at 20 and 40 minutes.

For the NB-UVA experiments, the LED array was placed 1cm from the surface of a culture plated with *E. coli* GFP, and illuminated (2000 μW/cm^2 at the plate). In separate experiments, we exposed liquid cultures of 10^6 CFU/mL of *E. coli* and *P. aeruginosa* to NB-UVA at intensities of 500, 1000, 2000 and 3000 μW/cm^2 for 20 and 40 minutes.

Safety of NB-UVA on human cells

HeLa cells (ATCC® CCL-2™) were added to DMEM cell culture medium (Gibco, Waltham, MA) plus 10% Bovine serum (Omega Scientific, Tarzana, CA) and 1x Antibiotic-Antimycotic (100x, Gibco) in 60x15mm standard tissue culture dishes (Corning, NY) and incubated at 37ºC (5% CO_2) for 24 hours to achieve 1,000,000 to 1,800,000 cells per plate. Cells were exposed to NB-UVA (2000 μW/cm^2) for 0 (control), 10, or 20min. After 24hr of further incubation at 37˚C (5% CO_2), cells were removed using 0.05% Trypsin-EDTA (1x) (Gibco), stained with Trypan Blue 0.4% (1:1) (Gibco) to define live/dead cells [13, 14] and quantitated using an automated cell counter (Biorad T20, Hercules, CA). HeLa cells were also exposed to higher NB-UVA at 5000 μW/cm^2 for 20 minutes and quantitated after 24hr of incubation at 37ºC (5% CO_2).

Effects of UVA were also tested on human alveolar (ATCC A549) and primary ciliated tracheal epithelial cells (HTEpC, Lot 446Z036.8, Male, age 50, Caucasian) (PromoCell, Heidelberg, Germany). Cells were plated and grown for 48h in DMEM (Alveolar cell) and Airway Growth Medium (HTEpC) (PromoCell) at 37ºC (5% CO_2). Subsequently, cells were exposed to UVA (2000 μW/cm^2) for 0 (control) or 20 minutes (treated), and cell counts were obtained after 24hr at 37ºC (5% CO_2) by automated cell counter (Biorad T20).

Levels of 8-hydroxy-2'-deoxyguanosineis (8-OHdG), a sensitive marker of oxidative DNA damage and oxidative stress [15, 16], were analyzed in the DNA of NB-UVA-treated cells. DNA was extracted using AllPrep DNA/RNA/Protein Mini Kits (Qiagen). 8-OHdG levels were detected using EpiQuik™ 8-OHdG DNA Damage Quantification Direct Kits (Epigentek, Farmingdale, NY). For optimal quantification, the input DNA amount was 300 ng, as the basal 8-OHdG is generally less than 0.01% of total DNA (Epigentek). A standard curve of 8-OHdG ranging from 5 to 200 pg was used to determine the concentration of 8-OHdG in the samples.

Effects of NB-UVA light on human cells transfected with group B coxsackievirus

NB-UVA exposure of HeLa cells transfected with group B coxsackievirus. HeLa cells were cultured (12 plates, mean 253,000 cells/plate) for 24hr at 37ºC (5% CO_2). Recombinant coxsackievirus B (pMKS1) expressing an enhanced green fluorescent protein (EGFP-CVB) was prepared as previously described [17]; half were exposed to NB-UVA (2000 μW/cm^2) for 20min while the other half were not exposed. HeLa cells were then transfected with NB-UVA-exposed or NB-UVA-unexposed virus (multiplicity of infection (MOI) = 0.1). Coxsackievirus is considered highly lytic [18]. After 6hrs, supernatant was removed, and cells were washed twice with 1x sterile PBS (pH = 7.0). New DMEM media was added and cells were incubated at 37ºC (5% CO_2). Dead cells in the supernatant (floating cells) were collected and quantified 24hrs later using an automated cell counter (Biorad T20). Six plates (3 NB-UVA-exposed and 3 unexposed) were assessed for live cells. Of the remaining six plates, the 3 plates transfected with NB-UVA-exposed virus were exposed to an additional 20min of NB-UVA (2000 μW/cm^2). After 24hrs at 37ºC (5% CO_2), imaging was performed using a BZ-9000 BioRevo (Keyence Corp., Itasca, IL). Dead and live cells were determined by the Trypan Blue 0.4% (1:1) (Gibco) method and counts were obtained using an automated cell counter (Biorad T20).

HeLa cell pre-treatment with NB-UVA and group B coxsackievirus transfection. HeLa cells were plated and incubated in DMEM for 24 hours at 37ºC (5% CO_2). Plates were divided into unexposed controls (n = 3) and HeLa cells exposed to NB-UVA (2000 μW/cm^2) for 20min (n = 3). After 24hrs at 37ºC (5% CO_2), all plates were transfected with EGFP-CVB (MOI = 0.1). At 24hrs post-transfection, cells were counted using an automated cell counter (Biorad T20).

Pre-treatment of group B coxsackievirus with NB-UVA and HeLa cell transfection. HeLa cells were cultured for 24hrs at 37ºC (5% CO_2) and transfected with EGFP-CVB (MOI = 0.1). Prior to transfection, half of the EGFP-CVB aliquots were exposed to NB-UVA (2000 μW/cm^2) and the other half remained unexposed. After 24hrs at 37ºC (5% CO_2), imaging was performed and HeLa cell counts were using an automated cell counter (Biorad T20).

Effects of repeated exposure of NB-UVA on HeLa cells already transfected with group B coxsackievirus. HeLa cells were plated and incubated at 37ºC (5% CO_2) and at 24hrs, cells were divided into three groups: Group 1, cells transfected with EGFP-CVB (n = 3, MOI = 0.1), served as positive transfected controls. Group 2, HeLa cells transfected with EGFP-CVB (MOI = 0.1) exposed to NB-UVA (n = 3, 2000 μW/cm^2 for 20 min) and 6hrs later exposed again to NB-UVA (2000 μW/cm^2) for 20 minutes followed by 4 additional exposures (two 20-minute exposures on day 2, 8hrs apart, and two 20-minute exposures on day 3, 8hrs apart. Group 3, not transfected with EGFP-CVB but exposed to NB-UVA at the same time-points as Group 2 (n = 3) to assess UVA effects. In all experiments, imaging and cell counts were performed using an automated cell counter (Biorad T20).

NB-UVA exposure on alveolar (A549) cells already transfected with group B coxsackievirus. Ideal timepoints of cell death from transfection were determined to be 24 hours in preliminary experiments with alveolar cells (results not shown). Alveolar cells were plated, incubated at 37ºC (5% CO_2) and counted at 48hrs (n = 9, cell count of 754,000). Cells were then transfected with EGFP-CVB (n = 6, MOI = 0.1), and 24hrs later, plated cells were exposed to NB-UVA (2000 μW/cm^2) for 0 (control, n = 3) or 20 minutes (treated, n = 3). Exposure was repeated every 24hrs for three days, with imaging and cell counts performed at 96hrs post-transfection. Three control plates were not transfected and not exposed.

Preparation of coronavirus 229E. Human coronavirus 229E (CoV-229E) (ATCC VR-740, ATCC) was overlain onto confluent MRC-5 human lung fibroblasts. CoV-229E is considered lytic [19]. Once cells exhibited ~50% cytopathic effect, cells were trypsinized and the cell/media suspension was collected. The cell/media mixture underwent one rapid freeze/thaw cycle and was centrifuged at 1000x g for 10min to clarify the media. The virus in the supernatant was used for subsequent experiments. Equal volumes of the supernatant from the same culture containing the virus were used for transfection of primary human cells.

NB-UVA exposure of ciliated tracheal epithelial cells (HTEpC) transfected with CoV-229E. HTEpC (135,000 cells) were plated into three groups. Group 1 was transfected with CoV-229E (n = 3, 50uL per plate). In group 2, prior to transfection, CoV-229E was exposed to NB-UVA (n = 3, 2000 μW/cm^2) for 20min. Group 3 was not exposed to NB-UVA or transfected (n = 3). After transfection, the cells were exposed to NB-UVA (4cm distance with 2000 μW/cm^2 at the plate surface) for 20min daily. Plates were imaged at 16, 36, 72, and 96hrs, cell viability (live/dead) counts were obtained at 72 and 96hrs post-transfection. Trypan Blue 0.4% (1:1) (Gibco) was used to determine live/dead cells and cell counts were obtained using an automated cell counter (Biorad T20, Hercules, CA). Cells were kept at 37ºC (5% CO_2).

NB-UVA effects on CoV-229E and mitochondrial antiviral signaling protein (MAVS). AllPrep DNA/RNA/Protein Mini Kits (Qiagen) were used to extract total protein from UVA-exposed and unexposed tracheal cells transfected with CoV-229E. Proteins were loaded into a Bolt 4–12% Bis-Tris gel (NW04122 Thermo Fisher) and transferred onto a Biotrace NT nitrocellulose membrane (27376–991, VWR). Total proteins were stained with Ponceau S solution (P7170, Sigma-Aldrich). The membrane was blocked in blocking solution (tris-buffered saline containing 3% bovine serum albumin (A7030, Sigma-Aldrich) and 0.1% Tween 20 (P1379, Sigma-Aldrich) (TBS-T) and incubated overnight at 4˚C with either rabbit anti-coronavirus spike protein antibody (1:1000; PA5-81777, Thermo Fisher) or mouse anti-MAVS antibody (1:200; SC-166583, Santa Cruz Biotechnology) diluted in blocking solution. After

washing in TBS-T, the membrane was then overlain with either horseradish peroxidase (HRP)-conjugated goat anti-rabbit IgG antibody (1:300; 95058–734, VWR) or HRP-conjugated goat anti-mouse IgG antibody (1:300; 5220–0286, SeraCare), washed in TBS-T, and exposed to enhanced chemiluminescence solution (RPN2235, GE Healthcare). Immunoreactive protein bands were imaged using a ChemiDoc Imaging System (Bio-Rad Laboratories, Hercules, CA).

In vivo effects of UVA

Animal preparation. *In vivo* effects of UVA exposure on mammalian internal visceral cells were assessed using wildtype 129S6/SvEv mice (n = 20, female = 10) and BALB/cJ mice (n = 10, female = 5). Animals were anesthetized prior to UVA light treatment in a chamber containing isoflurane anesthetic gas (1–5%) mixed with oxygen, and maintained under sedation using a nose cone anesthesia (1–2% isoflurane) at one breath per second. Euthanasia was performed using $C0_2$ inhalation followed by cervical dislocation. All animal research was performed under a protocol approved by the Institutional Animal Care and Use Committee (IACUC) at Cedars-Sinai Medical Center, IACUC007304.

Exposure of colonic mucosa to UVA. Under anesthesia, the borosilicate rod (OD = 4mm, length = 40mm) was introduced anally to the splenic flexure (S1C Fig). Five BALB/cJ mice underwent colonic BB-UVA exposure (2,000 μW/cm^2) for 30min, and 5 mice were treated with an unlit optic rod. In the second experiment, ten 129S6/SvEv mice underwent 20min daily colonic UVA exposure (3,000–3,500 μW/cm^2) for 2 two consecutive days, and 10 mice (male = 5) were treated with an unlit rod.

Endoscopic examination before and after UVA light therapy. While anesthetized, a rigid pediatric cystoscope (Olympus A37027A) was used to assess the intestinal mucosa up to the splenic flexure before and after UVA exposure. All endoscopies were recorded and blindly interpreted by two gastroenterologists (JHP and SYK) with expertise in animal model endoscopies. Endoscopic appearances were analyzed based on perianal examination, transparency of the intestinal wall, mucosal bleeding, and focal lesions.

Tissue analysis. At day 14, control and treated mice were euthanized, and swiss-roll preparations of the colon were performed as described [20]. The rolled colon was transferred to a tissue-processing/embedding cassette and placed in 10% buffered formalin overnight. Paraffin sections of the colon were cut, stained with hematoxylin and eosin (H&E), and assessed by a blinded pathologist (SS).

Statistical analysis

Descriptive statistics were calculated to describe the bacteria counts and colony sizes and UVA exposure with varying intensities. Each UVA group included 4 measurements and the mean of the measurements at each time point was reported. To assess the effect of UVA light on bacteria, yeast, and virus, the measurements of bacteria and human cells in UVA exposed and control groups were compared with t-test. Bivariate analyses were used to further determine the association between UVA exposure and viral effect on three human cell types. The continuous variables were compared with t-test. The statistical significance was defined as $p < 0.05$. Analyses were performed using GraphPAD Prism 7 (GraphPad, San Diego, CA).

Results

UVA effectively reduces bacteria and yeast

Exposure to BB-UVA (wavelength range 325-400nm) was associated with a significant microbial reduction at various time-points (Table 1). *E. coli* and *Pseudomonas aeruginosa* exposure

Table 1. Effect of broad band UVA light on bacteria and yeast based on time of exposure.

Microorganism	UVA Intensity (μW/cm^2)	Group	Baseline	20 min	% Reduction	Log Reduction	P value	40 min	% Reduction	Log Reduction	P value	60min	% Reduction	Log Reduction	P value
			CFUx10^7/ mL	CFUx10^7/ mL				CFUx10^7/ mL				CFUx10^7/ mL			
Clostridioides difficile	2,000	Exposed	0.10	0.08	38.46%	0.21	0.01	0.01	94.12%	1.23	0.003	0.0031	99.21%	2.10	0.01
		Control	0.10	0.13				0.17				0.39			
Candida albicans	1,700	Exposed	0.14	0.09	47.06%	0.28	0.007	0.03	85.00%	0.82	0.001	0.0032	98.00%	1.70	0.001
		Control	0.14	0.17				0.20				0.16			
Pseudomonas aeruginosa	3,500	Exposed	0.81	0.07	88.52%	0.94	<0.001	No Growth	*	*	<0.001	No Growth	*	*	<0.001
		Control	0.81	0.61				0.93				0.85			
Klebsiella pneumoniae	1,300	Exposed	5.90	6.34	15.35%	0.07	0.17	3.34	68.19%	0.50	<0.001	1.53	88.92%	0.96	<0.001
		Control	5.90	7.49				10.50				13.81			
Escherichia coli	1,300	Exposed	1.25	0.41	87.61%	0.91	<0.001	0.21	95.00%	1.30	0.001	0.03	99.46%	2.26	<0.001
		Control	1.25	3.31				4.20				5.52			
Enterococcus faecalis	2,400	Exposed	9.21	2.99	71.79%	0.55	0.1	0.61	95.86%	1.38	0.01	0.08	99.55%	2.35	0.01
		Control	9.21	10.60				14.72				17.74			
Streptococcus pyogenes	1,800	Exposed	1.17	0.68	18.07%	0.09	0.64	0.64	61.90%	0.42	0.001	0.17	87.02%	0.89	0.004
		Control	1.17	0.83				1.68				1.31			
Proteus mirabilis	2,400	Exposed	0.62	No Growth	*	*	<0.001	No Growth	*	*	<0.001	No Growth	*	*	<0.001
		Control	0.62	0.59				0.49				0.54			
Staphylococcus epidermidis	2,150	Exposed	0.57	0.43	27.12%	0.14	0.01	0.03	95.65%	1.36	<0.001	0.000117	99.98%	3.78	<0.001
		Control	0.57	0.59				0.69				0.7			

* No growth observed. Bactericidal threshold of >99.9% or 3 log reduction is met.

https://doi.org/10.1371/journal.pone.0236199.t001

Table 2. Effect of narrow-band UVA light on bacteria based on time exposure using varying intensities at 1 cm from the target.

Microorganism	UVA Intensity ($\mu W/cm^2$)	Group	Baseline CFUx10^7/mL	20 min CFUx10^7/mL	% Reduction	Log Reduction	P value	40 min CFUx10^7/mL	% Reduction	Log Reduction	P value
Escherichia coli GFP	500	Exposed	0.25	0.16	32.87	0.17	0.26	0.13	53.80	0.34	0.06
		Control	0.25	0.24				0.28			
	1000	Exposed	0.14	0.12	20.97	0.10	0.38	0.08	55.50	0.35	0.01
		Control	0.14	0.15				0.18			
	2000	Exposed	0.26	0.14	41.96	0.24	0.17	0.04	86.77	0.88	0.01
		Control	0.26	0.23				0.30			
	3000	Exposed	0.19	0.02	89.09	0.96	<0.001	No growth	*	*	0.01
		Control	0.19	0.15				0.19			
Pseudomonas aeruginosa	500	Exposed	0.49	0.27	24.79	0.12	0.17	0.24	-23.87	-0.09	0.47
		Control	0.49	0.36				0.19			
	1000	Exposed	0.49	0.24	25.24	0.13	0.01	0.10	37.07	0.20	<0.001
		Control	0.49	0.40				0.32			
	2000	Exposed	0.28	0.06	69.07	0.51	0.17	0.03	78.37	0.67	0.05
		Control	0.28	0.20				0.14			
	3000	Exposed	0.40	0.07	77.42	0.65	<0.001	0.02	89.23	0.97	0.01
		Control	0.40	0.32				0.22			

* No growth observed. Bactericidal threshold of >99.9% or 3 log reduction is met.

https://doi.org/10.1371/journal.pone.0236199.t002

to NB-UVA resulted in a significant dose-dependent reduction in bacterial cells and colony sizes (Table 2, S2 Table, Fig 1). The optimal NB-UVA intensity to impact bacteria appeared to be between 2000 and 3000μW/cm^2 at 1 cm from the target for 20 to 40 minutes (Table 2, S2 Table). The effect of NB-UVA on bacteria is a construct, with time of exposure, irradiance, distance from light source and wavelength all being important factors.

NB-UVA exposure is not associated with impaired cell viability or DNA damage. NB-UVA did not appear to affect growth of human cells tested (HeLa, alveolar A549 and tracheal epithelial cells). All plates demonstrated continued cell growth, regardless of UVA intensity, with 1.5 to 2 times the number of cells per plate compared to controls, indicating ongoing replication. NB-UVA did not affect HeLa cells growth and viability analyzed by trypan blue dye exclusion staining at 24hrs when compared to unexposed controls (10min of 2000 μW/cm^2 UVA: mean cell growth in treated 3.01x10^6 vs. mean cell growth untreated 2.3x10^6, $P = 0.53$; mean cell viability in treated 99.3% vs. mean cell viability in untreated 99.3%, $P > 0.99$; 20min of 2000 μW/cm^2 UVA: mean cell growth in treated 2.76x10^6 vs. mean cell growth in untreated 2.73x10^6, $P = 0.94$; mean cell viability in treated 99.6% vs. mean cell viability in untreated 99.3%, $P = 0.70$) (Fig 2A).

Higher intensity NB-UVA (5000 μW/cm^2) did not affect HeLa cells growth and viability (mean cell growth in treated 4.21x10^6 vs. mean cell growth in untreated 3.67x10^6, $P = 0.36$; mean cell viability in treated 99% vs. mean cell viability in untreated 99%, $P > 0.99$) (Fig 2B). Similar findings were observed with alveolar cells at 2000 μW/cm^2 for 20min (mean cell growth in treated 1.52x10^6 vs. mean cell growth in untreated 1.39x10^6, $P = 0.24$; mean cell viability in treated 97% vs. mean cell viability in untreated 99%, $P = 0.48$) (Fig 2C).

Finally, HTEpC growth and viability were also unaffected by NB-UVA after 20min of exposure to 1000 μW/cm^2 (mean cell growth in treated 1.41x10^5 vs. mean cell growth in untreated 1.26x10^5, $P = 0.61$; mean cell viability in treated 89.6% vs. mean cell viability in untreated 93.3%, $P = 0.173$) and to 2000 μW/cm^2 (mean cell growth in treated 1.83x10^5 vs. mean cell

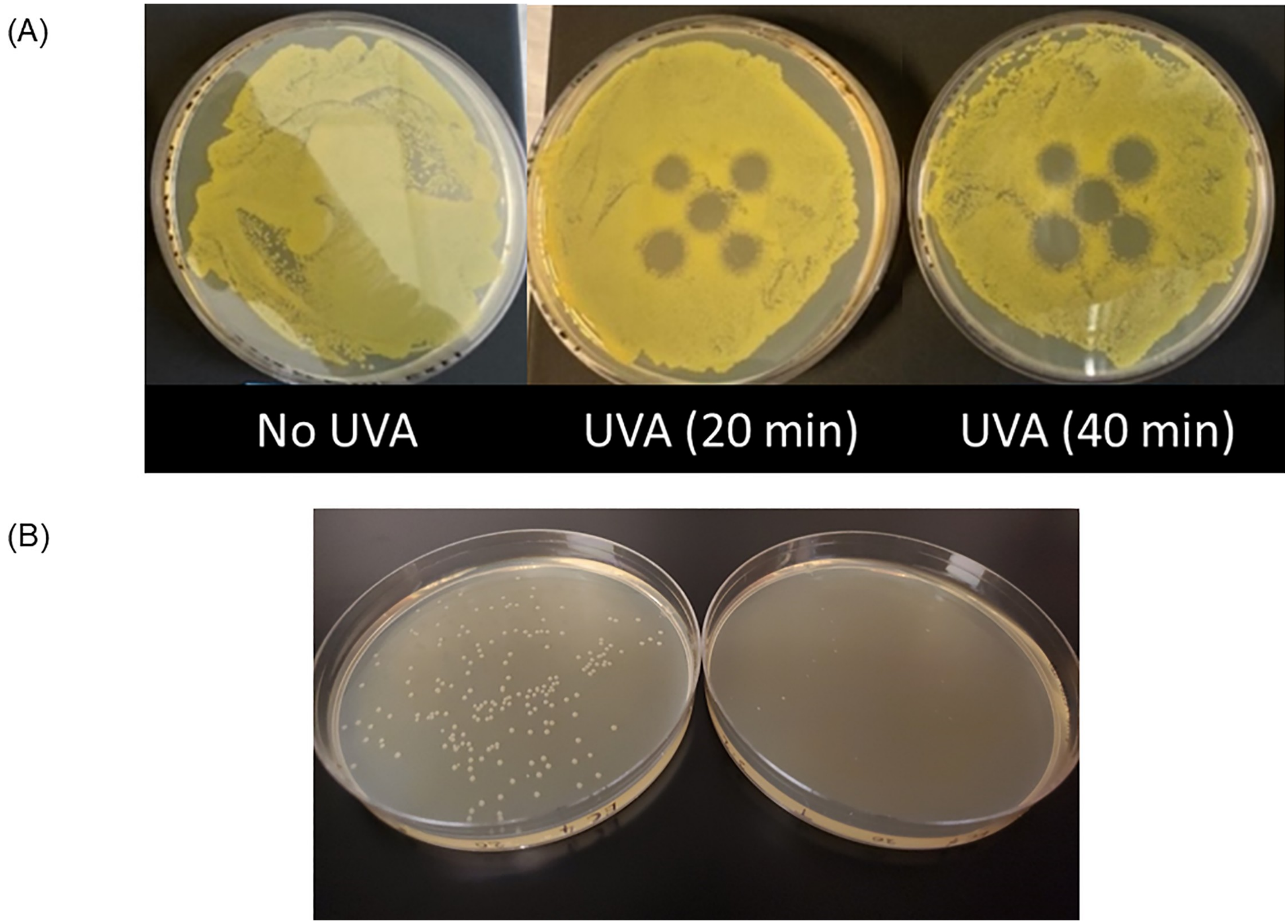

Fig 1. (A) Effect of narrow-band UVA exposure with intensity of 2000μW/cm^2 at the *E. coli* culture plate. An array of five LEDs was placed 1 cm above the plate, and plates were exposed for 20 and 40 minutes. (B) Effects of UVA treatment of *E. coli* liquid cultures when subsequently plated. Liquid *E. coli* cultures were treated with 3000 W/cm^2 for 20 minutes (right) or left untreated as controls (left), then plated on solid medium. There is a notable decrease in the number and size of *E. coli* colonies following UVA treatment.

https://doi.org/10.1371/journal.pone.0236199.g001

growth in untreated 2.18x10^5, P = 0.13; mean cell viability in treated 99% vs. mean cell viability in untreated 97%, P = 0.44).

Exposure to NB-UVA did not increase the levels of 8-OHdG in cells treated with NB-UVA as compared to controls (P>0.05) (Fig 3). Higher intensity NB-UVA (5000 μW/cm^2) appeared to increase 8-OHdG levels (P = 0.07) but the percentage of 8-OHdG remained well below the generally accepted safety threshold of 0.01% of the total DNA.

Effect of NB-UVA light on human cells transfected with group B coxsackievirus

EGFP-CVB UVA exposure prior to transfection of HeLa cells does not mitigate transfection. At 24 hours post-transfection, the percentage of dead cells was not different between plates with UVA pre-treated EGFP-CVB (62.0%±7.0) and untreated controls (69.0%±13.1) (P = 0.46); hence, pretreatment of EGFP-CVB did not appear to affect transfection of HeLa cells.

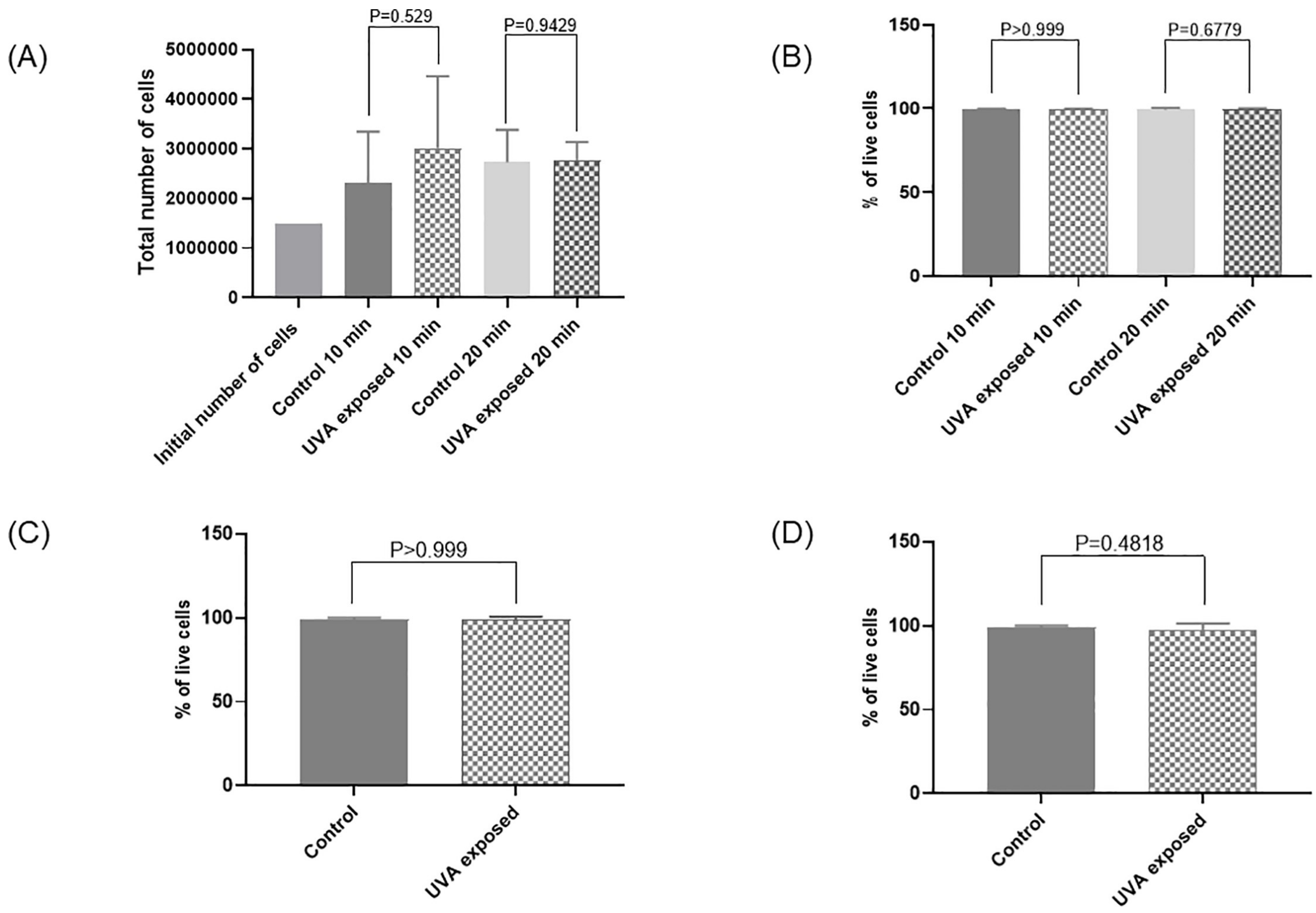

Fig 2. Effects of UVA treatment on cell growth and viability. (A) Effect of 10- and 20-minutes narrow-band (NB) UVA exposure (2000 μW/cm^2) on total number of HeLa cells (N = 3). (B) Effect of 10- and 20-minutes NB UVA exposure (2000 μW/cm^2) on percentage of viable HeLa cells (N = 3). (C) Effect of 20 minutes higher dose NB UVA exposure (5000 μW/cm^2) on percentage of viable HeLa cells (N = 3). (D) Effect of 20 minutes UVA exposure (2000 μW/cm^2) on percentage of viable alveolar cells (N = 3). Bars represent the mean value of the total number of cells (A) and percentage of live cells (B, C and D) for controls not exposed to UVA and cells exposed to UVA.

https://doi.org/10.1371/journal.pone.0236199.g002

HeLa cell exposure to UVA prior to EGFP-CVB transfection did not mitigate viral effects. In order to determine whether UVA pretreatment of cells affects the transfectivity of EGFP-CVB virus, half of the plates with HeLa cells were left untreated and the other half were pre-treated with 2000 μW/cm^2 NB-UVA for 20min. EGFP-CVB was added to both groups. Both groups were transfected, suggesting that pretreating HeLa cells with UVA prior to transfection did not influence the viral transfection rate.

NB-UVA exposure after transfection with EGFP-CVB reduces viral effects on HeLa cells. EGFP-CVB transfected HeLa cells were exposed to 2000 μW/cm^2 NB-UVA at 6hrs post-infection, then twice daily for two additional days, with cell counts at 72hrs. Transfected but UVA-unexposed cells served as controls. In the exposed group, UVA prevented cell death, with increased cell counts to 339,333 ± 60,781 at 72hrs (Fig 4), compared to no live cells remaining on plates at 48 (S2 Fig) and 72hrs in untreated controls. Importantly, HeLa cells that were not transfected but received NB-UVA exposure at the same time intervals showed normal cell proliferation, with cell counts of 2,413,333 ± 403,773 at 72hrs.

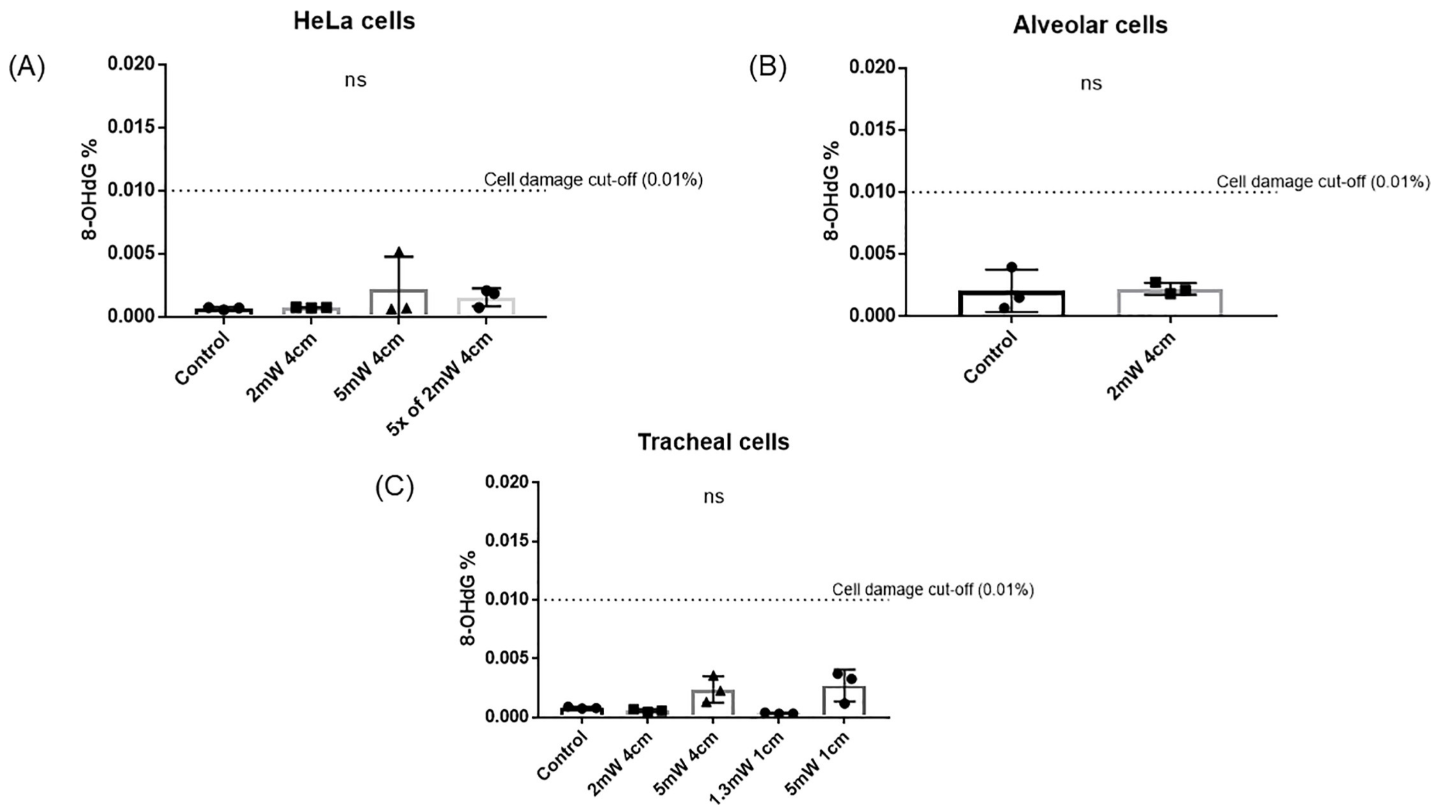

Fig 3. 8-OHdG levels after exposure to NB-UVA at various intensities at the cell plate on A) HeLa cells, B) alveolar cells and C) ciliated tracheal epithelial cells.

https://doi.org/10.1371/journal.pone.0236199.g003

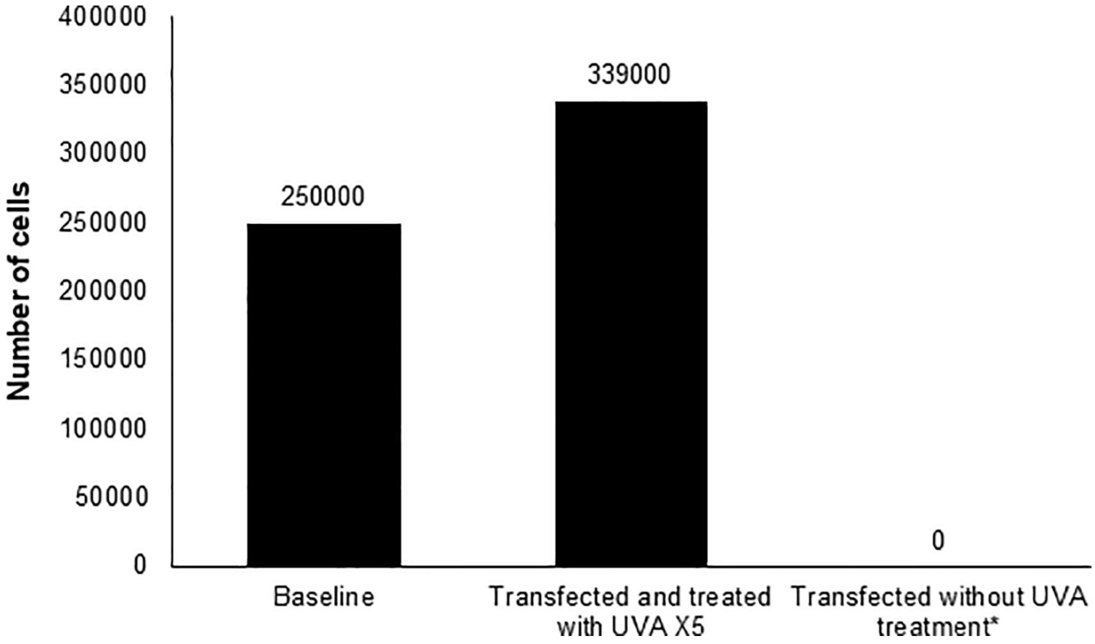

Fig 4. Effect of repeated UVA treatments on the number of adherent HeLa cells post-transfection with Coxsackievirus at 72 hours. *The number of adherent cells at 48 hours were below the limit of detection of the automated cell counter used (Biorad T20), and no adherent cells were observed at 72 hours.

https://doi.org/10.1371/journal.pone.0236199.g004

Effect of NB-UVA exposure on alveolar (A549) cells transfected with EGFP-CVB. Compared to HeLa cells, transfection with EGFP-CVB induced less death in alveolar cells. Alveolar cells treated with NB-UVA also demonstrated transfection, but visual assessment suggested lower rates of transfection, with fewer cells producing viral EGFP signals (S3 Fig). Viable cell counts were numerically higher in the UVA exposed group (90.0%±2.0) compared to the unexposed group (72.7%±23.9), however, statistical significance was not reached (P = 0.28)

NB-UVA light preserves tracheal cells transfected with CoV-229E. Transfected HTEpC produced definitive changes in cell morphology. However, non-transfected control cells and transfected cells treated with daily UVA exhibited predominantly normal morphology (Fig 5). At 96hrs, there were no differences in cell counts and viability between controls (mean cell number: 3.33×10^5, mean cell viability: 92±1.0%) and transfected UVA-treated cells (mean cell number: 3.48×10^5; mean cell viability: 95±2.5%) suggesting that UVA exposure prevented virus-induced cell death (cell number P = 0.88; cell viability: P = 0.16). In contrast, there was a significant reduction in viable cells in untreated transfected cells (mean cell number: 1.99×10^5; mean cell viability: 70±4.36%) compared to transfected UVA-treated cells (mean cell number: 3.48×10^5; mean cell viability: 95±2.5%) (cell number P = 0.02; cell viability: P = 0.001) (Fig 6A).

Transfected cells exposed to NB-UVA showed decreased CoV-229E spike (S) protein (~130kDa) when compared to transfected cells not treated with UV (Fig 6B). Moreover, cells transfected with CoV-229E and treated with UVA had increased levels of MAVS when compared to UVA-naïve cells transfected with CoV-229E, suggesting that UVA may activate MAVS to enter an antiviral state without triggering antiviral apoptotic cell death (Fig 6B).

Single or repeated colonic BB-UVA light exposure is not associated with endoscopic or histologic injury in mice

No perforation, bleeding or death were seen during intracolonic UVA exposure of mice. Endoscopic evaluation of mice before and after single or repeated UVA administration demonstrated no macroscopic evidence of mucosal erythema, friability, ulceration or bleeding (Fig 7A). Furthermore, no ulcerations, acute/chronic inflammation, cryptitis, crypt abscesses, granulomata, or dysplasia were identified under microscopic examination of full-thickness colonic specimens exposed UVA as compared to controls and untreated segments of the colon as assessed by a blinded pathologist (SS) (Fig 7B).

Discussion

The global health community is facing formidable challenges resulting from the emergence of novel pathogens and the rise of antimicrobial resistance. Internal UVA light may provide an inexpensive, effective solution. Treatment with UVA light at specific intensities, peak-wavelengths, exposure times and distances is effective against bacteria (*Pseudomonas aeruginosa*, *Klebsiella pneumoniae*, *Escherichia coli*, *Enterococcus faecalis*, *Clostridioides difficile*, *Streptococcus pyogenes*, *Staphylococcus epidermidis*, *Proteus mirabilis)*, yeast (*Candida albicans)* and viruses (Coronavirus-229E and Coxsackievirus B) *in vitro*. Supratherapeutic (5,000 μW/cm2 for 20 minutes) or repeated (up to five 20-minute sessions) exposure of human cells to UVA did not affect cell survival or significantly increase DNA oxidation. *In vivo*, single and repeated broad-spectrum intracolonic UVA exposure in mice did not show harm on endoscopic evaluation and full-thickness pathologic assessment when compared to unexposed control animals. Our findings suggest that controlled UVA may be effective against various pathogens, does not appear to harm human cells *in vitro*, and exhibits a favorable *in vivo* safety profile.

Increments in duration of exposure improved microbial reduction by UVA in our experiments (Tables 1 and 2). Reductions in colony count and size were statistically significant but

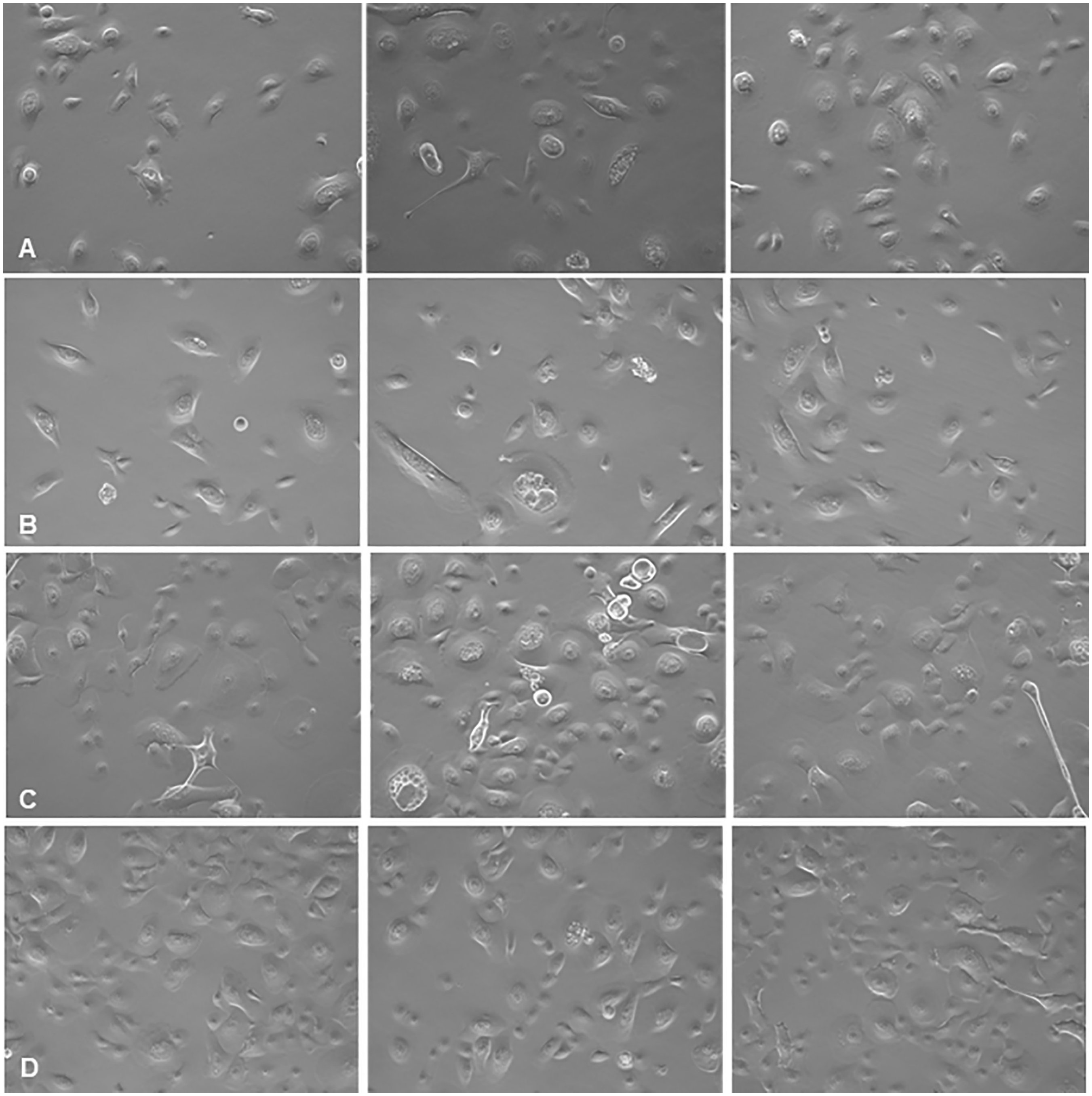

Fig 5. Effect of UVA treatment on coronavirus 229E infection of HTeC cells at A) 16 hours, B) 36 hours, C) 72 hours and D) 96 hours post transfection (20x phase-contrast images). Left panels: Uninfected, untreated control cells. Middle panels: cells transfected with coronavirus 229E. Right panels: cells transfected with UVA-treated coronavirus 229E and then treated with UVA. Cells transfected with coronavirus 229E exhibit increasing vacuolation and cell death over time, resulting in decreased cell density. In contrast, transfected and UVA-treated cells remain viable and exhibit similar morphology to controls.

https://doi.org/10.1371/journal.pone.0236199.g005

within the timeframe of UVA exposure in our experiments (60 minutes or less), the bactericidal threshold of 1000-fold reduction (i.e. >99.9% reduction) in bacterial density [21] was not met for several microbes. Mechanisms underlying the antimicrobial effects of UV light are not fully known. It is postulated that formation of pyrimidine(6–4)pyrimidone and cyclobutane

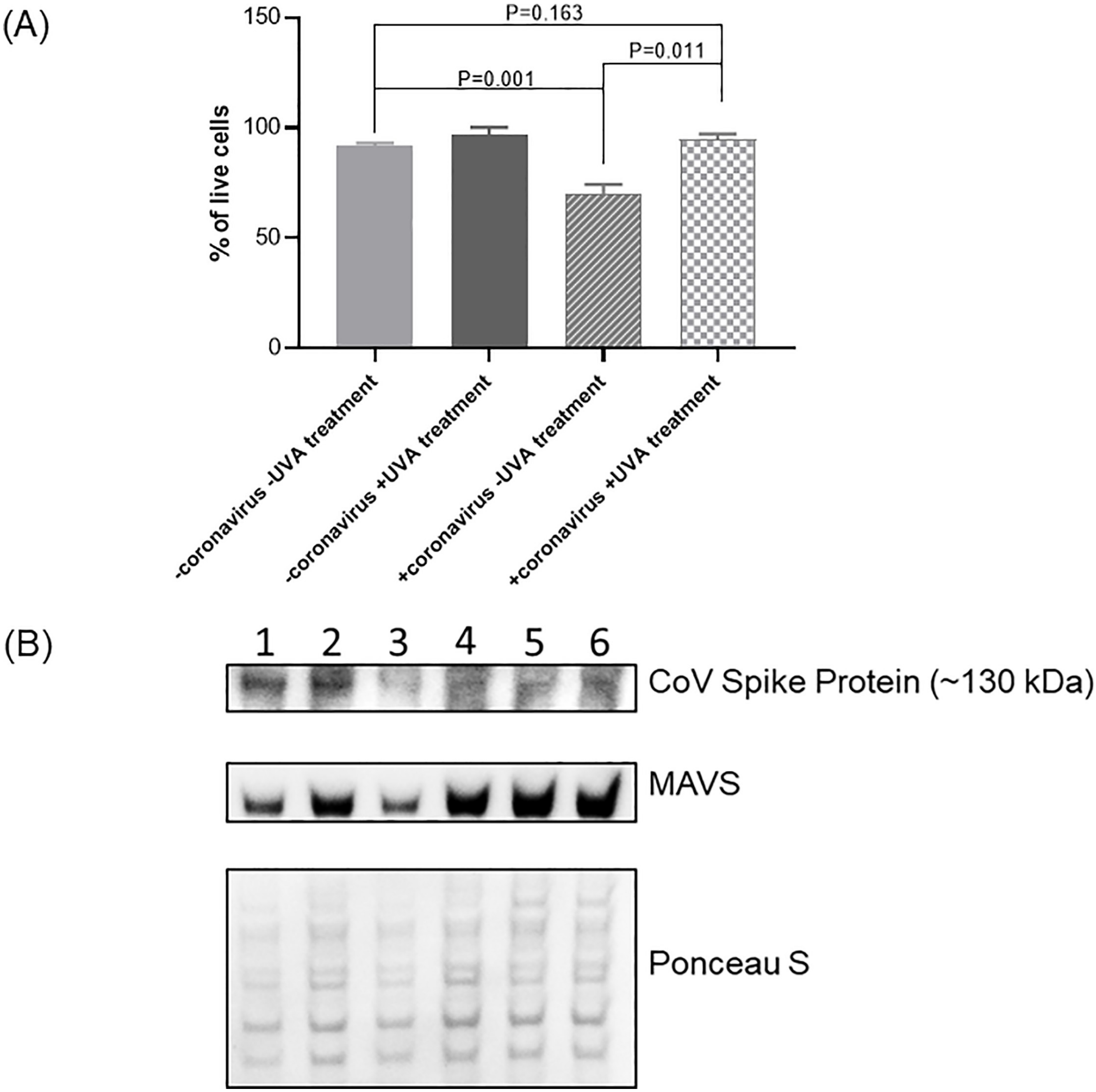

Fig 6. A) Viability of ciliated tracheal epithelial cells depending on transfection with coronavirus 229E and treatment with UVA light after 96 hours. Transfected ciliated tracheal epithelial cells showed 25% ±1.84 more viability when treated with NB UVA. B) Intracellular detection of coronavirus 229E in ciliated tracheal epithelial cells treated with NB-UVA light at 96 hours and levels of Mitochondrial antiviral-signaling protein (MAVS). Column 1, 2, and 3 represent cells transfected with CoV-229E; column 4, 5 and 6 –cells transfected with CoV-229E and treated with NB-UVA. Ponceau S Stain was used to locate overall protein bands to check the amount of protein loaded on the gel.

https://doi.org/10.1371/journal.pone.0236199.g006

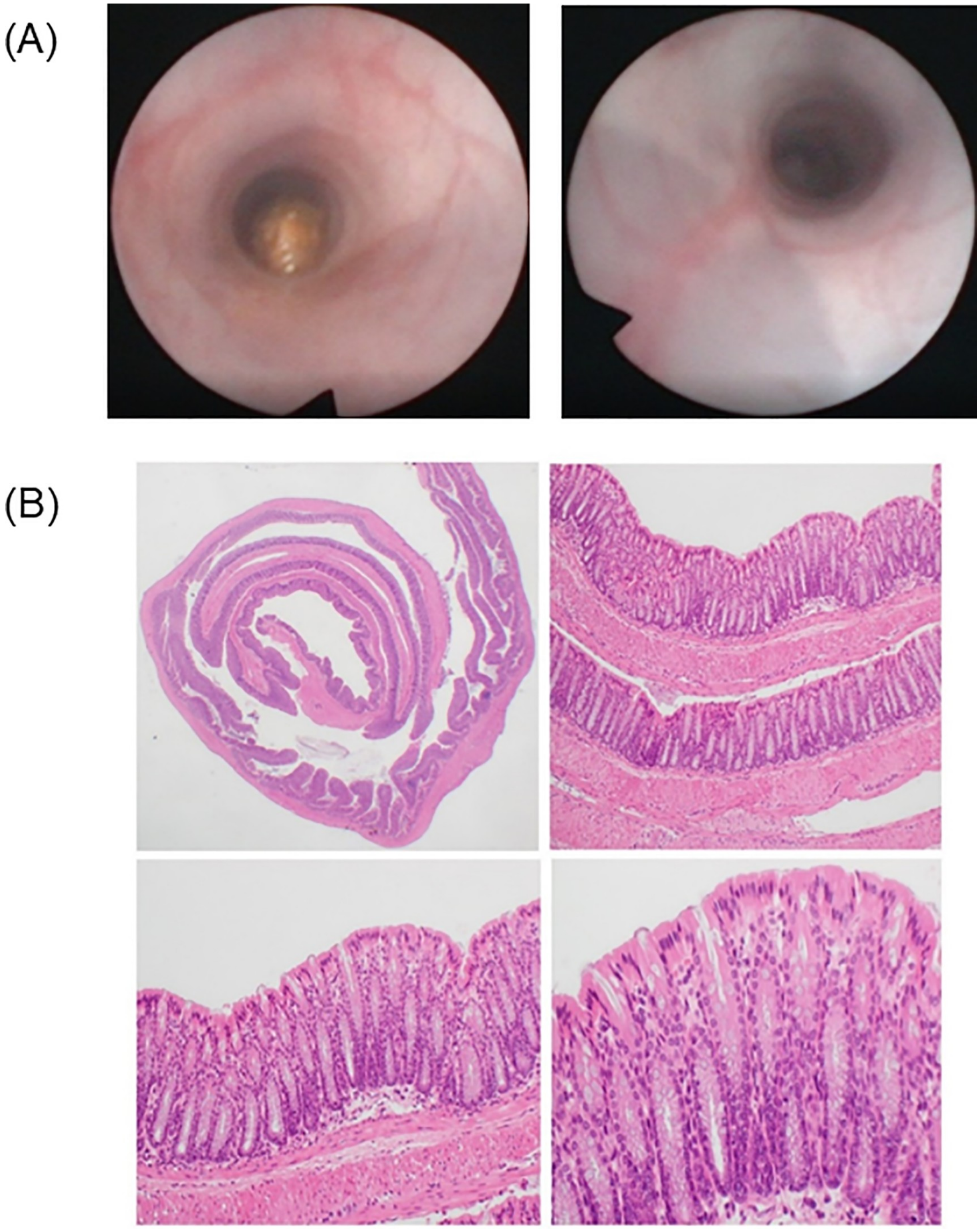

Fig 7. A) Colonoscopic images before and 72 hours after repeated UVA treatment in mice. B) Full-thickness histological examination of the post-mortem colon in BB-UVA-treated mice at various magnifications. Clockwise from top to left, H&E microscopic examinations at 12.5X, 100X, 200X and 400X magnification. There is no evidence of endoscopic or histologic abnormalities.

https://doi.org/10.1371/journal.pone.0236199.g007

pyrimidine dimers upon exposure to light disrupts microbial DNA and RNA, inhibiting replication [22]. For this reason, UV light has been associated with toxicity to human cells. While UVA therapy does not have known systemic toxicity [23], prolonged, high dose exposure to

UVA has been associated with melanoma [24, 25]. Our *in vitro* studies showed no growth retardation or excess DNA oxidation of HeLa, alveolar, and ciliated tracheal epithelial cells exposed to controlled doses of UVA. In addition, *in vivo* single or repeated intraluminal exposure was not associated with inflammation or dysplasia in the short term. These findings support the plausibility of controlled internal UVA exposure as an antimicrobial therapeutic modality. However, further elucidation of potential long-term toxicity is warranted. Of note, despite known harmful effects of UVB [26] and UVC including skin cancers [27, 28], short-term UVB therapy is an approved treatment for several inflammatory dermatological conditions [7], and far UVC light is being explored for its properties that include antimicrobial effects without harm to mammalian cells [29].

Application of internal UVA has several potential advantages. First, there is no evidence for *de novo* resistance to UV radiation against pathogens. Second, the onset of UVA antimicrobial effects may be significantly faster than existing therapies which may take days to reach peak anti-microbial effects. Our results show that 20 to 40 minutes of exposure to UVA is effective against pathogens. Third, internal UVA is now feasible. Traditionally, UV studies involved large incandescent, fluorescent or mercury vapor lamps [24] requiring significant distance from the target for heat protection and homogenization of UV irradiance. Miniature LEDs have improved stability, durability, thermal properties, and more precise peak wavelengths than traditional UV lamps [30]. Our results show that controlled narrow-band UVA emitted by LEDs in close proximity to the target appears to be effective against various pathogens.

An important finding in our study is that UVA exhibits antiviral effects against positive sense, single-stranded RNA viruses including coxsackievirus group B and coronavirus-229E. Furthermore, we examined whether antiviral signaling mechanisms were altered with UVA treatment, and found that MAVS protein increases after UVA exposure. This component of cellular antiviral immunity plays a central role in viral suppression [31] and suggests that UVA may activate MAVS to enter an antiviral state without triggering antiviral apoptotic cell death. The mechanism by which UVA induces MAVS overexpression requires further elucidation. Furthermore, various modes of cell death including apoptosis and necrosis need to be assessed in the future.

Our findings may support the clinical evaluation of internally applied UVA to treat or prevent pathogenic infections of the respiratory tract, digestive tract, and others. One immediate potential application may be to decrease SARS-CoV-2 viral load in severely affected patients. SARS-CoV-2 replicates in upper respiratory tract ciliated epithelial cells [32], which then shed and can lead to compromised pulmonary function [1, 33–35] and death [36]. Compromised pulmonary function warrants mechanical ventilation in 2–5% of infected individuals [1, 33–35, 37]. Ventilator-associated pneumonia (VAP), as a secondary microbial infection, has been reported in 31% of COVID-19 patients who require intubation [38]. Decreasing/delaying tracheal and endotracheal tube (ETT) colonization has been associated with lower rates of VAP [39, 40]. It is conceivable that controlled, intermittent UVA in the upper airway may reduce viral burden and/or prevent VAP. Clinical studies are warranted to assess antimicrobial effects of internal UVA phototherapy. To explore this, we first assessed whether UVA is a potential alternative to antibiotic and antifungal treatments. Intubated COVID19 patients suffer from high rate of VAP, which is caused by secondary bacterial or fungal infections. If UVA therapy is effective against these agents, it could potentially decrease morbidity and mortality in COVID19 patients due to VAP. Second, we aimed to show the utility of UVA in suppressing RNA viruses including human coronavirus 229E. Third, we assessed the safety of UVA for human cells including alveolar and tracheal cells *in vitro*. Fourth, as the effects of in-vivo internal UV therapy have not been investigated previously, we explored the safety of applying UVA light internally on murine colonic mucosa, as a first animal model of internal UVA therapy.

This model has several advantages over other intraluminal exposure models: 1) it allows for repeated UVA treatments under identical controlled conditions, 2) it permits *in vivo* assessment of the mucosa using endoscopy without the need for euthanasia, 3) the unexposed right colon serves as a histologic control for the exposed left colon in each animal, and 4) as shown in the control experiments, intracolonic placement of UV emitting devices does not lead to local or systemic injuries as a potential confounder to safety assessment of internal UV therapy.

Our study has several limitations. While multiple daily short-term UVA treatments did not harm human cells and appeared safe *in vivo*, longer term use may require further study. We assessed UVA against several microbes, but more studies are needed to address additional pathogens, including multi-drug resistant organisms, mycobacteria, and archaea. We did not evaluate UVA against SARS-CoV-2 specifically. However, given the efficacy of UVA against coxsackievirus and CoV-229 (both positive sense, single-stranded RNA viruses), SARS-CoV-2 is likely also UVA-sensitive.

In conclusion, under specific conditions, UVA reduces bacteria, fungi and viruses including CoV-229E, and in the short-term does not harm mammalian cells in preclinical studies. Determination of whether the reduction in bacterial and viral loads seen here have a clinical impact will require human *in vivo* testing. Controlled internal UVA therapy can potentially provide a safe and novel modality for treating human pathogens.

Supporting information

S1 Fig. A) Customized side-emitting borosilicate light rod used for broad-band UVA experiments. B) Narrow-band UVA LED light configuration. C) UVA light application in mice for *in vivo* safety determination. Right side: mouse undergoing colonic UVA therapy up to splenic flexure under anesthesia. Left side: control mouse exposed to unlit rod.
(DOCX)

S2 Fig. Effects of NB-UVA exposure on HeLa cells transfected with group B coxsackievirus. A) 24 hours after transfection: Reduced number of adherent cells in UVA-unexposed plates (left panel, percent dead cells in supernatant = 67.5±11.0%) compared with UVA-exposed plates (right panel, percent dead cells in supernatant = 16.1±5.8%) (P = 0.002).
(Magnification = 4x, overlay of green light and bright field).
(DOCX)

S3 Fig. Effects of NB-UVA exposure on alveolar (A549) cells transfected with EGFP-CVB Transfected alveolar cells treated with NB UVA exhibit less viral EGFP signals (left panel) when compared to transfected alveolar cells not treated with UVA (right panel)
(Magnification = 4x, overlay of green light and bright field).
(DOCX)

S1 Table. *In vitro* exposure of pathogens to UVA, including growth conditions, intensity, and duration of UVA exposures.
(DOCX)

S2 Table. Effect of NB-UVA light on bacterial colony diameter based on time exposure across varying intensities.
(DOCX)

S1 Raw Images.
(PDF)

Acknowledgments

We would like to thank Zev Melmed for his assistance in 3-D modeling, and Dr. Peter Chen for his help in study planning strategies for virology. We would especially like to thank the Gottlieb lab at Cedars-Sinai for allowing us to utilize their virology capabilities in these experiments. Dr. Ralph Feuer at San Diego State University for generously providing the EGFP-CVB viral plasmid used in these studies. We also thank Shreya Celly, Dr. Daniel Cohrs and Maya Pimentel for their assistance in a number of the bacterial culture experiments. Finally, we would like to thank Dr. Gillian Barlow from the Cedars-Sinai MAST Program for her assistance in editing the manuscript.

Author Contributions

Conceptualization: Ali Rezaie, Ruchi Mathur, Mark Pimentel.

Data curation: Ali Rezaie, Gabriela G. S. Leite, Maria Jesus Villanueva-Millan, Gonzalo Parodi, Jon Sin, Juliana F. Germano, Walter Morales, Stacy Weitsman, Seung Young Kim, Jae Ho Park.

Formal analysis: Ali Rezaie, Gabriela G. S. Leite, Gil Y. Melmed, Ruchi Mathur, Siamak Sakhaie, Mark Pimentel.

Funding acquisition: Ali Rezaie.

Investigation: Ali Rezaie, Gabriela G. S. Leite, Maria Jesus Villanueva-Millan, Gonzalo Parodi, Jon Sin, Juliana F. Germano, Walter Morales, Stacy Weitsman, Seung Young Kim, Jae Ho Park, Siamak Sakhaie.

Methodology: Gabriela G. S. Leite, Gil Y. Melmed, Maria Jesus Villanueva-Millan, Gonzalo Parodi, Jon Sin, Juliana F. Germano, Walter Morales, Stacy Weitsman, Seung Young Kim, Jae Ho Park, Siamak Sakhaie.

Project administration: Ali Rezaie, Ruchi Mathur, Mark Pimentel.

Supervision: Ali Rezaie, Walter Morales, Stacy Weitsman, Mark Pimentel.

Writing – original draft: Ali Rezaie, Gabriela G. S. Leite, Gil Y. Melmed, Ruchi Mathur, Maria Jesus Villanueva-Millan, Walter Morales, Siamak Sakhaie, Mark Pimentel.

Writing – review & editing: Ali Rezaie, Gabriela G. S. Leite, Gil Y. Melmed, Ruchi Mathur, Mark Pimentel.

References

1. Huang C, Wang Y, Li X, Ren L, Zhao J, Hu Y, et al. Clinical features of patients infected with 2019 novel coronavirus in Wuhan, China. Lancet. 2020; 395(10223):497–506. Epub 2020/01/28. https://doi.org/10.1016/S0140-6736(20)30183-5 PMID: 31986264.
2. World Health Organization. Ultraviolet radiation (UV) [cited 2020]. Available from: https://www.who.int/uv/uv_and_health/en/#:~:text=The%20UV%20region%20covers%20the,(100%2D280%20nm).
3. World Health Organization. Protecting Workers from Ultraviolet Radiation. World Health Organization; 2007.
4. Reed NG. The history of ultraviolet germicidal irradiation for air disinfection. Public Health Rep. 2010; 125(1):15–27. Epub 2010/04/21. https://doi.org/10.1177/003335491012500105 PMID: 20402193; PubMed Central PMCID: PMC2789813.
5. Roy S. Impact of UV Radiation on Genome Stability and Human Health. Adv Exp Med Biol. 2017; 996:207–19. Epub 2017/11/11. https://doi.org/10.1007/978-3-319-56017-5_17 PMID: 29124702.

6. Bhutani T, Liao W. A Practical Approach to Home UVB Phototherapy for the Treatment of Generalized Psoriasis. Pract Dermatol. 2010; 7(2):31–5. Epub 2010/08/01. PMID: 25191138; PubMed Central PMCID: PMC4151182.
7. Zhang P, Wu MX. A clinical review of phototherapy for psoriasis. Lasers Med Sci. 2018; 33(1):173–80. Epub 2017/10/27. https://doi.org/10.1007/s10103-017-2360-1 PMID: 29067616; PubMed Central PMCID: PMC5756569.
8. Patrizi A, Raone B, Ravaioli GM. Safety and Efficacy of Phototherapy in the Management of Eczema. Adv Exp Med Biol. 2017; 996:319–31. Epub 2017/11/11. https://doi.org/10.1007/978-3-319-56017-5_27 PMID: 29124712.
9. Tarabadkar ES, Shinohara MM. Skin Directed Therapy in Cutaneous T-Cell Lymphoma. Front Oncol. 2019; 9:260. Epub 2019/04/30. https://doi.org/10.3389/fonc.2019.00260 PMID: 31032224; PubMed Central PMCID: PMC6470180.
10. Reichrath J, Rass K. Ultraviolet damage, DNA repair and vitamin D in nonmelanoma skin cancer and in malignant melanoma: an update. Adv Exp Med Biol. 2014; 810:208–33. Epub 2014/09/11. https://doi.org/10.1007/978-1-4939-0437-2_12 PMID: 25207368.
11. Robatto M, Pavie MC, Garcia I, Menezes MP, Bastos M, Leite HJD, et al. Ultraviolet A/blue light-emitting diode therapy for vulvovaginal candidiasis: a case presentation. Lasers Med Sci. 2019; 34 (9):1819–27. Epub 2019/04/05. https://doi.org/10.1007/s10103-019-02782-9 PMID: 30945022.
12. Lahuerta Zamora L, Perez-Gracia MT. Using digital photography to implement the McFarland method. J R Soc Interface. 2012; 9(73):1892–7. Epub 2012/02/18. https://doi.org/10.1098/rsif.2011.0809 PMID: 22337631; PubMed Central PMCID: PMC3385748.
13. Strober W. Trypan blue exclusion test of cell viability. Curr Protoc Immunol. 2001;Appendix 3:Appendix 3B. Epub 2008/04/25. https://doi.org/10.1002/0471142735.ima03bs21 PMID: 18432654.
14. Louis KS, Siegel AC. Cell viability analysis using trypan blue: manual and automated methods. Methods Mol Biol. 2011; 740:7–12. Epub 2011/04/07. https://doi.org/10.1007/978-1-61779-108-6_2 PMID: 21468962.
15. Kasai H. Analysis of a form of oxidative DNA damage, 8-hydroxy-2'-deoxyguanosine, as a marker of cellular oxidative stress during carcinogenesis. Mutat Res. 1997; 387(3):147–63. Epub 1998/01/24. https://doi.org/10.1016/s1383-5742(97)00035-5 PMID: 9439711.
16. Pilger A, Rudiger HW. 8-Hydroxy-2'-deoxyguanosine as a marker of oxidative DNA damage related to occupational and environmental exposures. Int Arch Occup Environ Health. 2006; 80(1):1–15. Epub 2006/05/11. https://doi.org/10.1007/s00420-006-0106-7 PMID: 16685565.
17. Taylor DJR, Hamid SM, Andres AM, Saadaeijahromi H, Piplani H, Germano JF, et al. Antiviral Effects of Menthol on Coxsackievirus B. Viruses. 2020; 12(4). Epub 2020/04/02. https://doi.org/10.3390/v12040373 PMID: 32231022.
18. Feuer R, Mena I, Pagarigan R, Slifka MK, Whitton JL. Cell cycle status affects coxsackievirus replication, persistence, and reactivation in vitro. J Virol. 2002; 76(9):4430–40. Epub 2002/04/05. https://doi.org/10.1128/jvi.76.9.4430-4440.2002 PMID: 11932410; PubMed Central PMCID: PMC155066.
19. Tang BS, Chan KH, Cheng VC, Woo PC, Lau SK, Lam CC, et al. Comparative host gene transcription by microarray analysis early after infection of the Huh7 cell line by severe acute respiratory syndrome coronavirus and human coronavirus 229E. J Virol. 2005; 79(10):6180–93. Epub 2005/04/29. https://doi.org/10.1128/JVI.79.10.6180-6193.2005 PMID: 15858003; PubMed Central PMCID: PMC1091719.
20. Bialkowska AB, Ghaleb AM, Nandan MO, Yang VW. Improved Swiss-rolling Technique for Intestinal Tissue Preparation for Immunohistochemical and Immunofluorescent Analyses. J Vis Exp. 2016;(113). Epub 2016/08/09. https://doi.org/10.3791/54161 PMID: 27501188; PubMed Central PMCID: PMC4993444.
21. Wald-Dickler N, Holtom P, Spellberg B. Busting the Myth of "Static vs Cidal": A Systemic Literature Review. Clin Infect Dis. 2018; 66(9):1470–4. Epub 2018/01/03. https://doi.org/10.1093/cid/cix1127 PMID: 29293890; PubMed Central PMCID: PMC5905615.
22. Vatansever F, Ferraresi C, de Sousa MV, Yin R, Rineh A, Sharma SK, et al. Can biowarfare agents be defeated with light? Virulence. 2013; 4(8):796–825. Epub 2013/09/27. https://doi.org/10.4161/viru.26475 PMID: 24067444; PubMed Central PMCID: PMC3925713.
23. Lapolla W, Yentzer BA, Bagel J, Halvorson CR, Feldman SR. A review of phototherapy protocols for psoriasis treatment. J Am Acad Dermatol. 2011; 64(5):936–49. Epub 2011/03/25. https://doi.org/10.1016/j.jaad.2009.12.054 PMID: 21429620.
24. Khan AQ, Travers JB, Kemp MG. Roles of UVA radiation and DNA damage responses in melanoma pathogenesis. Environ Mol Mutagen. 2018; 59(5):438–60. Epub 2018/02/22. https://doi.org/10.1002/em.22176 PMID: 29466611; PubMed Central PMCID: PMC6031472.

25. Viros A, Sanchez-Laorden B, Pedersen M, Furney SJ, Rae J, Hogan K, et al. Ultraviolet radiation accelerates BRAF-driven melanomagenesis by targeting TP53. Nature. 2014; 511(7510):478–82. Epub 2014/06/12. https://doi.org/10.1038/nature13298 PMID: 24919155; PubMed Central PMCID: PMC4112218.

26. Takada A, Matsushita K, Horioka S, Furuichi Y, Sumi Y. Bactericidal effects of 310 nm ultraviolet light-emitting diode irradiation on oral bacteria. BMC Oral Health. 2017; 17(1):96. Epub 2017/06/08. https://doi.org/10.1186/s12903-017-0382-5 PMID: 28587675; PubMed Central PMCID: PMC5461700.

27. Ichihashi M, Ueda M, Budiyanto A, Bito T, Oka M, Fukunaga M, et al. UV-induced skin damage. Toxicology. 2003; 189(1–2):21–39. Epub 2003/06/25. https://doi.org/10.1016/s0300-483x(03)00150-1 PMID: 12821280.

28. Zaffina S, Camisa V, Lembo M, Vinci MR, Tucci MG, Borra M, et al. Accidental exposure to UV radiation produced by germicidal lamp: case report and risk assessment. Photochem Photobiol. 2012; 88 (4):1001–4. Epub 2012/03/31. https://doi.org/10.1111/j.1751-1097.2012.01151.x PMID: 22458545.

29. Ponnaiya B, Buonanno M, Welch D, Shuryak I, Randers-Pehrson G, Brenner DJ. Far-UVC light prevents MRSA infection of superficial wounds in vivo. PLoS One. 2018; 13(2):e0192053. Epub 2018/02/22. https://doi.org/10.1371/journal.pone.0192053 PMID: 29466457; PubMed Central PMCID: PMC5821446.

30. Shen L, Griffith TM, Nyangaresi PO, Qin Y, Pang X, Chen G, et al. Efficacy of UVC-LED in water disinfection on Bacillus species with consideration of antibiotic resistance issue. J Hazard Mater. 2020; 386:121968. Epub 2020/01/03. https://doi.org/10.1016/j.jhazmat.2019.121968 PMID: 31896007.

31. Biacchesi S, LeBerre M, Lamoureux A, Louise Y, Lauret E, Boudinot P, et al. Mitochondrial Antiviral Signaling Protein Plays a Major Role in Induction of the Fish Innate Immune Response against RNA and DNA Viruses. J Virol. 2009; 83(16):7815. https://doi.org/10.1128/JVI.00404-09 PMID: 19474100

32. Chan JF, Yuan S, Kok KH, To KK, Chu H, Yang J, et al. A familial cluster of pneumonia associated with the 2019 novel coronavirus indicating person-to-person transmission: a study of a family cluster. Lancet. 2020; 395(10223):514–23. Epub 2020/01/28. https://doi.org/10.1016/S0140-6736(20)30154-9 PMID: 31986261.

33. Xu Z, Shi L, Wang Y, Zhang J, Huang L, Zhang C, et al. Pathological findings of COVID-19 associated with acute respiratory distress syndrome. Lancet Respir Med. 2020; 8(4):420–2. Epub 2020/02/23. https://doi.org/10.1016/S2213-2600(20)30076-X PMID: 32085846.

34. Chen N, Zhou M, Dong X, Qu J, Gong F, Han Y, et al. Epidemiological and clinical characteristics of 99 cases of 2019 novel coronavirus pneumonia in Wuhan, China: a descriptive study. Lancet. 2020; 395 (10223):507–13. Epub 2020/02/03. https://doi.org/10.1016/S0140-6736(20)30211-7 PMID: 32007143.

35. Shi H, Han X, Jiang N, Cao Y, Alwalid O, Gu J, et al. Radiological findings from 81 patients with COVID-19 pneumonia in Wuhan, China: a descriptive study. Lancet Infect Dis. 2020; 20(4):425–34. Epub 2020/02/28. https://doi.org/10.1016/S1473-3099(20)30086-4 PMID: 32105637; PubMed Central PMCID: PMC7159053.

36. Grasselli G, Zangrillo A, Zanella A, Antonelli M, Cabrini L, Castelli A, et al. Baseline Characteristics and Outcomes of 1591 Patients Infected With SARS-CoV-2 Admitted to ICUs of the Lombardy Region, Italy. JAMA. 2020. Epub 2020/04/07. https://doi.org/10.1001/jama.2020.5394 PMID: 32250385; PubMed Central PMCID: PMC7136855.

37. Murthy S, Gomersall CD, Fowler RA. Care for Critically Ill Patients With COVID-19. JAMA. 2020. Epub 2020/03/12. https://doi.org/10.1001/jama.2020.3633 PMID: 32159735.

38. Zhou F, Yu T, Du R, Fan G, Liu Y, Liu Z, et al. Clinical course and risk factors for mortality of adult inpatients with COVID-19 in Wuhan, China: a retrospective cohort study. Lancet. 2020; 395(10229):1054–62. Epub 2020/03/15. https://doi.org/10.1016/S0140-6736(20)30566-3 PMID: 32171076.

39. Kollef MH, Afessa B, Anzueto A, Veremakis C, Kerr KM, Margolis BD, et al. Silver-coated endotracheal tubes and incidence of ventilator-associated pneumonia: the NASCENT randomized trial. JAMA. 2008; 300(7):805–13. Epub 2008/08/21. https://doi.org/10.1001/jama.300.7.805 PMID: 18714060.

40. McGlave P. Bone marrow transplants in chronic myelogenous leukemia: an overview of determinants of survival. Semin Hematol. 1990; 27(3 Suppl 4):23–30. Epub 1990/07/01. PMID: 1695763.
